# Integrated multi-dimensional micro-structure reveals the changes in moisture migration and quality attributes during drying of apple slices with ultrasonic pretreatment

**DOI:** 10.1016/j.fochx.2025.103168

**Published:** 2025-10-21

**Authors:** Cheng Zhang, Zina Lin, Xiaolong Li, Jiachi Duan, Jiaqi Liu, Shuang Luo, Liangyu Dai, Bing Tian, Jun Wang, Jun Li

**Affiliations:** a School of Public Health/Key Laboratory of Environmental Pollution Monitoring and Disease of Ministry of Education, Guizhou Medical University, Guian, 561113, China; b College of Food Science and Engineering, Northwest A&F University, Yangling 712100, China; c Guizhou Agricultural Ecology and Resource Protection Station, Agriculture and Rural Affairs Department of Guizhou Province, Guiyang 550001, China

**Keywords:** Multi-dimensional micro-structure, Ultrasonic pretreatment, Drying characteristics, Cell wall polysaccharide, Ultrastructure, Gallic acid (PubChem CID: 370), Protocatechuic acid (PubChem CID: 72)., Chlorogenic acid (PubChem CID: 1794427)., Caffeic acid (PubChem CID: 689043)., Epicatechin (PubChem CID: 72276)., Ferulic acid (PubChem CID: 445858)., Ellagic acid (PubChem CID: 5281855)., Oxalic acid (PubChem CID: 971)., Citric acid (PubChem CID: 311)., Malic acid (PubChem CID: 525).

## Abstract

Optimizing drying efficiency and enhancing product quality are crucial for industrial apple dehydration processing. This study focused on multi-dimensional micro-structure changes of apple slices with ultrasonic pretreatment (US). The results indicated that US pretreatment significantly enhanced mass transfer by depolymerizing cell wall pectin to increase water-soluble pectin (72.15 to 90.35 mg/g AIR) and chelate-soluble pectin (35.31 to 54.69 mg/g AIR), converting bound water to free water, disrupting the structural integrity of the cells, thereby reducing drying time by 13.31–38.82 % for hot-air drying and 32.60–36.88 % for infrared drying. Concurrently, the microscopic structural changes induced by US treatment enhanced rehydration capacity and minimized bio-active compound's loss, particularly chlorogenic acid and malic acid. Although the reduction in total phenols and flavonoids resulted in decreased antioxidant activity, appropriate US treatment enhanced color by activating polyphenol oxidase (PPO). Consequently, optimal US pretreatment is essential for enhancing both the drying efficiency and quality of apple slices.

## Highlights

• Ultrasonic pretreatments accelerate moisture migration of apple slices.
• Ultrasonic induced depolymerization of polysaccharide from apple cell wall.
• Ultrasound enhances key metabolites retention in dried apples.
• Optimal ultrasonic pretreatment boosts apple slice drying efficiency and quality.

## 1 Introduction

The perishable nature of food items contributes to post-harvest losses. Minimizing post-harvest food losses and prolonging the shelf life of edible items are crucial enhancing global food security in the face of climate change and population growth (Usama et al., 2023). Therefore, drying, the oldest method for extending the shelf life of crops, has been widely by food processors as the initial unit operation in the food processing chain. However, the main challenge is to preserve the nutritional and aesthetic quality of food while minimizing energy consumption (Benhamza et al., 2021).

As the global food industry has rapidly expanded, food drying has also seen rapid development. However, drying is an energy-intensive processing method, and statistics show that it accounts for more than 30 % of the total energy consumption in the food processing industry (Chojnacka et al., 2021). As global energy demand continues to rise, the issue of energy scarcity is becoming increasingly prominent. Simultaneously, the excessive carbon emissions, environmental pollution, and economic burden associated with high energy consumption have become major concerns (Corigliano & Algieri, 2024). Therefore, energy conservation and emission reduction have become core priorities for the processing and manufacturing industry, including the food drying sector.

The composition of food raw materials is complex and their structures are diverse, which can have varying degrees of influence on moisture removal during the drying process. During drying, as the moisture content decreases, the material undergoes internal shrinkage and its original microstructure changes. This hinders the formation of water migration channels, reduces the rate of water migration, prolongs drying time, and increases energy consumption (Maskan, 2001). Furthermore, the aggregation state of food matrices, such as polysaccharides, proteins, and starch, undergoes changes during the drying process. This alters their binding force with water and reduces the rate of water migration (Zhang, Ding, et al., 2024). Therefore, auxiliary improvements in food structure and the formation of an organizational structure that facilitates water migration can effectively enhance drying efficiency and reduce energy consumption during drying (Llavata et al., 2025).

The cavitation effect of ultrasonic waves can enhance the formation of a honeycomb structure inside the food, facilitating the migration of internal water and the dissolution of nutrients (Azoubel et al., 2010; Chen et al., 2020). However, excessive ultrasonic treatment can lead to nutrient loss, which adversely affects the quality retention of the dried product (Sun et al., 2024). Critically, the developed internal microstructure plays a dual role, it regulates water diffusion pathways for efficient removal, yet its integrity is paramount to prevent leaching and degradation of valuable components. Achieving a balance with moderate ultrasonic assistance is crucial for improving drying efficiency while maximizing nutrient retention. The analysis of material microstructure serves as a key bridge in this regard.

Dehydration represents a critical processing technique for apple slices, serving as a primary strategy to mitigate post-harvest spoilage losses. Nevertheless, conventional drying processes induce significant quality deterioration in apple slices, characterized by thermal degradation of l-ascorbic acid, volatilization of key aroma compounds, and oxidation of bio-active polyphenols (Bai et al., 2022; Li et al., 2019). Concurrently, the substantial energy demand associated with prolonged dehydration elevates production costs and environmental impacts. Thus, developing targeted pretreatment strategies to concurrently enhance retention of thermolabile nutrients, preserve organoleptic properties, and reduce specific energy consumption holds compelling scientific and industrial relevance. This study aimed to investigate the effects of different ultrasonic pretreatment times (0 min, 10 min, 20 min, and 30 min) and drying methods on the drying characteristics and quality of apple slices. It focused on the degradation of cell wall polysaccharides and the changes in the microstructure during the pretreatment process to elucidate the drying-promoting mechanism of ultrasonic pretreatment and its influence on the quality characteristics of the slices (color, primary secondary metabolites, and antioxidant properties). This research provides a theoretical foundation for the application of ultrasonic pretreatment technology to enhance drying efficiency, save energy, and maintain quality in fruits and vegetables.

## 2 Materials and method

### 2.1 Raw materials

The apple samples were sourced from the high-altitude late-maturing cultivar production region in Heishitou Town (Weining Yi, Hui and Miao Autonomous County), located in the Bijie City of Guizhou Province, Southwest China. This unique cultivation area (2000–2400 m above sea level) features distinctive agroclimatic conditions characterized by: mean annual temperature of 15 °C; frost-free period averaging 198 days; annual solar exposure of 1777.5 h; total solar radiation reaching 110.5 kcal/cm^2^; and substantial annual precipitation averaging 1086 mm. The combination of elevated terrain, prolonged photo-period, and significant thermal accumulation creates optimal growing conditions for delayed fruit maturation and phytochemical development. The initial moisture content of the apples was determined via oven-drying method at 105 °C for 24 h, yielding a value of 86.93 ± 0.16 g/100 g (wet basis). Selected specimens with intact morphology and undamaged epidermis underwent sequential processing: washing, manual peeling, and transverse sectioning into 3-mm thick slices using a mechanical slicer, followed by core removal. Subsequently, the sliced apple tissues were formed into concentric circular rings (30 mm diameter) using a precision cutting apparatus. These uniform rings were quartered into four geometrically identical segments for subsequent ultrasonic pretreatment and drying experiments.

### 2.2 Ultrasonic pretreatments and drying

#### 2.2.1 Ultrasonic pretreatments

The freshly prepared apple slices underwent immersion in a color-preservation solution containing 1 % (*w*/*v*) citric acid and 2 % (w/v) ascorbic acid for 10 min, maintaining a controlled mass ratio of 1:10 (sample:solution). Ultrasonic pretreatment using a 20 kHz generator system at 400 W was no more than 35 °C maintained by ice-bath cooling (Kahraman et al., 2021; Wu et al., 2020), with treatment durations of 0, 10, 20, and 30 min establishing temporal gradients. Post-treatment samples were subjected to comparative dehydration through two distinct thermal processing methods: hot-air drying (HD) and infrared drying (IR), with operational temperatures set at 50, 60, and 70 °C. The dehydration process was terminated upon reaching target moisture content thresholds (12–15 % wet basis), after which the processed specimens were equilibrated in desiccators for subsequent analytical procedures.

#### 2.2.2 Drying methods

Based on extensive preliminary experiments and literature research (Önal et al., 2019), the hot-air drying (HD) procedure was implemented using an electric convection dryer (Model WGL—B, Tester Instrument Co., Ltd., Tianjin, China) under controlled thermal conditions of 50, 60, and 70 °C (Wang et al., 2022). The system maintained a constant air velocity of 1.5 m/s through precision fan regulation. Comparative infrared drying (IR) trials were performed in a dedicated infrared dehydration chamber (Taizhou Senttech Infrared Technology Co., Ltd., Jiangsu, China) operated at identical temperature gradients (50–70 °C) (Wang et al., 2022), utilizing quartz heating elements with standardized power output of 1350 W. Both drying regimens incorporated real-time temperature monitoring via integrated PID control systems to ensure process stability. Throughout the drying trials, samples were loaded at the maximum design capacity of the drying systems. The effective drying area measured 1.2 m^2^ for HD and 0.8 m^2^ for IR, with a uniform material loading density of 20.25 kg/m^2^ maintained across all treatments. Drying was continued until the mass changes of samples were within 0.05 g of the last three recorded values (Wang et al., 2022).

#### 2.2.3 Drying kinetics

The moisture ratio (*MR*) vs the time curve was obtained based on Eq. (1).

(1) $$\mathrm{MR}=\frac{\mathrm{Mt}-\mathrm{Me}}{M0-\mathrm{Me}}$$

where, *MR* is the moisture ratio of samples, *M*_*t*_ (g water/g dry solid) is the moisture content of samples at drying time t, *M*_*0*_ (g water/g dry solid) is the initial moisture content, *M*_*e*_ (g water/g dry solid) is the equilibrium moisture content. Since *M*_*e*_ < <*M*_*0*_, the effect of *M*_*e*_ can be ignored, Eq. (1) can be simplified to Eq. (2).

(2) $$\mathrm{MR}=\frac{\mathrm{Mt}}{M0}$$

The drying rate (*DR*) of apple slices was calculated according to Eq. (3).

(3) $$\mathrm{DR}=\frac{\mathrm{Mt}1-\mathrm{Mt}2}{t2-t1}$$

where *t*_*1*_ and *t*_*2*_ is the drying time (h), *M*_*t1*_ and *M*_*t2*_ are the dry basis moisture content at *t*_*1*_ and *t*_*2*_, g/g.

### 2.3 Multi-dimensional micro-structure observation

#### 2.3.1 Cell structure observation

The cellular ultrastructure of ultrasonically treated apple slices was characterized using an inverted fluorescence microscope (IX73, Olympus, Tokyo, Japan) with bright field imaging capabilities, following modified methodologies from Bao et al. (2024). Cross-sectional specimens (10 μm thickness) were prepared from the mesocarp tissue using a cryostat microtome (CM1860, Leica Biosystems, Nussloch, Germany) at −20 °C. Bright field imaging was conducted under standardized laboratory conditions (23 °C, 50 % RH) using white light illumination at 200× magnification.

#### 2.3.2 Observation of cell wall ultrastructure

The cellular ultrastructure of apple parenchyma tissue was characterized through transmission electron microscopy (TEM) analysis. Tissue specimens (initially excised as 1 mm × 3 mm pieces) excised from fresh apple mesocarp were processed according to modified protocols from Ben-Arie and Kislev (1979). Primary fixation was performed in 2.5 % (*v*/v) glutaraldehyde solution prepared in 0.1 M acetate buffer (pH 7.2) for 2 h at 4 °C, followed by three 15-min buffer rinses. Secondary fixation employed 1 % (*w*/*v*) osmium tetroxide in identical buffer conditions for 2 h, with subsequent buffer washing cycles repeated quadruplicately. Gradual dehydration was achieved through an ethanol series (35, 50, 70, 90, 95, and 100 % v/v) with 15-min immersion per concentration.

Dehydrated samples were sequentially infiltrated with propylene oxide and progressively increasing concentrations of spurr's epoxy resin (1:1, 1:2, and pure resin). Final embedding was conducted in silicone molds through 24-h polymerization at 60 °C following 12-h resin penetration at 4 °C. Ultrathin sections (70–90 nm) were obtained using a diamond knife-equipped ultramicrotome (Leica EM UC7), mounted on 200-mesh copper grids, and stained with uranyl acetate/lead citrate. TEM imaging was performed using a JEOL JEM-1400Flash instrument operated at 120 kV, with systematic image acquisition of ≥10 non-overlapping fields per biological replicate.

#### 2.3.3 Cell-wall polysaccharide fractions and ultrastructure measurement

The sequential extraction of alcohol-insoluble residue (AIR) and its constituent pectin fractions including water-soluble pectin (WSP), chelate-soluble pectin (CSP), and sodium carbonate-soluble pectin (NSP) from apple parenchyma tissue was conducted according to literature research (Li et al., 2022).

Pectin content was quantified via the meta-hydroxydiphenyl colorimetric assay (Wang et al., 2021), with galacturonic acid (GalA) equivalents calculated from absorbance values at 520 nm. Cellulose and hemicellulose contents were determined using the anthrone‑sulfuric acid method (Wang et al., 2021), with glucose as the calibration standard (620 nm detection). All analyses included three independent biological replicates with technical triplicates.

Transmission electron microscopy (TEM; HT7800, Hitachi High-Tech, Tokyo, Japan) was employed to analyze pectin nanostructures. Pectin fractions (0.05 mg/mL in ultrapure water) were thermally equilibrated at 50 °C for 1 h in a water bath. Aliquots (1 μL) were deposited onto 300-mesh carbon-coated copper grids (Ted Pella Inc.) and air-dried for 12 h under controlled humidity (40 % RH). Imaging was performed at 100 kV accelerating voltage, with representative micrographs captured from ≥5 non-overlapping fields per sample.

### 2.4 Texture analysis

The textural properties of apple samples were evaluated using a texture analyzer (TA.XT Plus, Stable Micro Systems) following a modified protocol adapted from Zhang et al. (2015). Compression tests were performed with a cylindrical probe (P/5, 5 mm diameter) under controlled parameters: 50 % strain deformation at a constant crosshead speed of 0.50 mm/s, with a 2.0-s inter-cycle interval. Ten spatially distributed measurement points were randomly selected per treatment group to ensure representative sampling. Force-time curves were recorded using Texture Exponent 32 software (v6.1.16), with textural parameters calculated from triplicate biological replicates.

### 2.5 Determination of water binding state

Ultrasound (US)-pretreated apple slices were subjected to transverse relaxation time (T₂) analysis using a benchtop NMR analyzer (Niumag PQ001–20-025 V, 0.5 T, Shanghai Niumag Corp., China). Post-treatment specimens were equilibrated to ambient temperature (25 °C ± 1 °C) prior to loading into 25 mm NMR-grade borosilicate tubes. Real-time T₂ measurements were initiated within 30 s of sample loading using a 15-mm RF coil maintained at 32.0 °C ± 0.5 °C via Peltier temperature control. The Carr-Purcell-Meiboom-Gill (CPMG) pulse sequence was executed with optimized parameters: 90° pulse width = 18 μs, 180° pulse width = 36 μs, inter-echo spacing (τ) = 500 ms, and 8192 echoes acquired per scan. Magnetic field homogeneity was verified daily using deuterium oxide standards. Triplicate measurements per biological replicate were performed with 2-min thermal equilibration between consecutive runs.

### 2.6 Determination of color and polyphenol oxidase

#### 2.6.1 Surface color

Surface color parameters of dehydrated apple slices were quantified using a calibrated chromatic analyzer (CM-5, Konica Minolta Sensing, Shanghai, China) in CIE Lab^⁎^ color space. The tri-stimulus values were recorded as *L*^⁎^ (Luminosity index, 0–100 scale; 100 = pure white, 0 = absolute black), *a*^⁎^ (Red-green chromatic coordinate, −60 to +60; positive = red hue, negative = green hue), and *b*^⁎^ (Yellow-blue chromatic coordinate, −60 to +60; positive = yellow chromaticity, negative = blue chromaticity). The cumulative color variation induced by processing treatments was calculated through total color difference (*ΔE*) according to Eq. (3) (Zambrano-Zaragoza et al., 2014):

(4) $$\mathrm{\Delta Ε}=\sqrt{{\left(L^{*}-L0^{*}\right)}^{2}+{\left(a^{*}-a0^{*}\right)}^{2}+{\left(b^{*}-b0^{*}\right)}^{2}}$$

where *L*₀^⁎^, *a*₀^⁎^, and *b*₀ ^⁎^represent the chromatic values of fresh untreated controls. Measurements were conducted under D65 illuminant with specular component excluded mode.

#### 2.6.2 Assessment of PPO activity

PPO extraction was performed following modified protocols from Yeoh and Ali (2017). Fresh apple tissue (2.0 ± 0.1 g) from non-ultrasonicated controls and US-treated groups (10, 20, 30 min) was homogenized with 15 mL ice-cold 0.1 M phosphate buffer (pH 6.5) using a 1:7.5 (*w*/*v*) ratio in a pre-chilled mortar. The homogenate was centrifuged (CR22GIII, Hitachi, Japan) at 10,000 ×*g* for 10 min at 4 °C. Supernatants were double-filtered through 0.45 μm nylon membranes (Millipore), pooled, and maintained at 4 °C for subsequent analysis within 2 h. PPO activity quantification employed a spectrophotometric method (Krystian et al., 2018) with the following reaction system: 0.2 mL 0.1 M phosphate buffer (pH 6.5), 0.1 mL 50 mM catechol substrate (Sigma-Aldrich), 0.3 mL crude enzyme extract. Absorbance kinetics were monitored at 420 nm using a UV–Vis spectrophotometer (UV-2600, Shimadzu) equipped with a thermostatted cell holder (25 °C). Initial absorbance (T₀) was recorded immediately post-mixing, followed by T₃ measurement after 180 s incubation. Blank controls replaced enzyme extract with buffer. Enzyme activity units (U) were defined as ΔA₄₂₀/min × 10^3^, where ΔA = (A₃ - A₀) - (A_blank₃_ - A_blank₀_) (Wang et al., 2019).

### 2.7 Kinetics of rehydration and scanning electron microscope observation

The rehydration ratio (RR) was employed to evaluate cellular integrity loss during dehydration. Dried apple slices (1.50 ± 0.05 g) were subjected to controlled rehydration following an optimized protocol adapted from Yang et al. (2020) and preliminary experiments. Samples were immersed in 75 mL distilled water (25.0 ± 0.5 °C) under static conditions for 120 min, maintaining a 1:50 (*w*/*v*) solid-to-liquid ratio. At 20-min intervals, specimens were retrieved using stainless steel mesh baskets, surface moisture was blotted with qualitative filter paper, and mass recorded via analytical balance. The RR was calculated as:

(5) $$\mathrm{RR}=\frac{\mathrm{Wr}-\mathrm{Wd}}{\mathrm{Wd}}$$

where *W*_*d*_ represents the initial dry mass, *W*_*r*_ represents the rehydrated mass.

Dehydrated apple tissues were sputter-coated with 10 nm gold‑palladium (Q150T ES, Quorum Technologies) prior to scanning electron microscopy (SEM) analysis (S-3400 N, Hitachi High-Tech, Tokyo, Japan). Secondary electron imaging was performed at 15 kV accelerating voltage under high vacuum (10^−3^ Pa), with working distance maintained at 10.0 mm. Representative micrographs were acquired at 50× and 250× magnifications using an integrated digital imaging system.

### 2.8 Phytochemical and antioxidant determination

#### 2.8.1 Total phenol

Total phenolic content (TPC) was determined using the Folin–Ciocalteu method (Xu et al., 2015) with minor modifications. In brief, 0.5 mL of the diluted samples (1:20) was combined with 1 mL of Folin–Ciocalteu reagent and allowed to react for 3 min at room temperature. Subsequently, 4 mL of Na_2_CO_3_ (120 g/L) was added to the mixture. The absorbance at 765 nm was measured after incubating the solution in the dark for 60 min. The TPCs were expressed as gallic acid equivalent (GAE) (mg GAE/g DM).

#### 2.8.2 Total flavonoids

Total flavonoid content (TFC) of the dried samples was measured using a previously reported method (Gowd et al., 2016). Initially, 2 mL of diluted samples (1:10) was mixed with 0.3 mL of NaNO_2_ (5 % *w*/*v*) and allowed to react for 5 min. Subsequently, 0.3 mL of AlCl_3_ (10 % w/v) was added to the mixture and allowed to react for 6 min. Next, 2 mL of NaOH (4 % w/v) was added to the mixture. The absorbance at 510 nm was measured after 15 min. The results were expressed as rutin equivalents (RE) (mg RE/100 g DM).

#### 2.8.3 Organic acids

The extract was evaporated to dryness under reduced pressure (35 °C, 50 mbar) using a rotary evaporator (Model DLSB-5/20, Gongyi Yuhua Instrument Co., Ltd., Henan, China). The resultant residue was reconstituted with HPLC-grade methanol to a final volume of 10 mL. Prior to analysis, the solution was filtered through a 0.45 μm polytetrafluoroethylene (PTFE) syringe filter. Chromatographic separation was performed on an HPLC system (LC-20 A, Shimadzu Corp., Kyoto, Japan) equipped with a diode array detector, with simultaneous monitoring at 280 nm and 320 nm, following the chromatographic conditions described by Tunc (2025).

Organic acid quantification was conducted using the aforementioned HPLC system according to the methodology established by Kossah et al. (2009). Individual analytes were quantified by external calibration curves constructed using authentic standards. Calibration curves for all target compounds were prepared using standard solutions across a concentration range of 0.1–100 μg/mL, achieving correlation coefficients (R^2^) ≥0.998 (Supplementary material Table S2). Data were normalized to sample dry weight and expressed as milligrams of organic acid per gram of dry matter (mg/g DM).

#### 2.8.4 Monomer phenol

The concentrations of ten monomeric phenols in apple flakes were determined using high-performance liquid chromatography (HPLC) with minor modifications, as described by Li et al. (2019). A 10 mL polyphenol extract was concentrated using a rotary evaporator at 35 °C under reduced pressure. The resulting residue was dissolved in methanol and adjusted to a final volume of 10 mL for chromatography. Subsequently, the solution was filtered through a 0.45 μm nylon membrane prior to HPLC analysis. The chromatographic column employed was an Inert Sustain C18 (4.6 mm × 250 mm, 5 μm). The chromatographic conditions consisted of mobile phase A, which was a 1 % aqueous solution of formic acid, and mobile phase B, which was acetonitrile. The gradient elution procedure was as follows: 0–5 min at 5 % B; 5–25 min at 12 % B; 25–40 min at 30 % B; 40–50 min at 45 % B; and 50–60 min at 5 % B. The flow rate was maintained at 1 mL/min, the column temperature at 30 °C, the sample volume at 10 μL, and the detection wavelength at 280 nm. Chromatography-grade monomeric phenols were utilized as standards, and the results were expressed as milligrams per 100 g of dry matter (DM). Table S2 presents the standard curves for each phenolic standard.

#### 2.8.5 In vitro antioxidant

The antioxidant activities of the dried apple samples were evaluated using the DPPH radical scavenging activity (RSA) and ferric reducing antioxidant power (FRAP) assays. The DPPH RSA method was modified with minor adjustments, as described by Li et al. (2022). The sample extracts were diluted to twice their original volume, and 0.5 mL of the diluted solution was subsequently added to 5 mL of an ethanolic solution containing DPPH at a concentration of 0.1 mmol/L. The solution was incubated in the dark for 30 min, and the absorbance (A) at 517 nm was measured using a UV-VIS spectrophotometer (UV-1780, Shimadzu Instruments Co., Ltd., Suzhou, China), with ethanol serving as the control. The DPPH RSA (%) can be calculated using the following formula:

The method for determining the ferric reducing antioxidant power (FRAP) of dried samples was modified with minor adjustments based on a previous study by Sethi et al. (2020). Briefly, 0.1 mL of the diluted samples (1:5) was combined with 2 mL of distilled water and 3 mL of the working FRAP reagent. Subsequently, the mixture was incubated in the dark for 30 min, and the absorbance was measured at 593 nm, with distilled water serving as the control. The results are expressed as millimoles of FeSO_4_ equivalents per milligram of dry mass (DM).

### 2.9 Data analysis

The data are presented as the mean ± standard deviation (SD) of three replicates, with all substance concentrations reported on a dry mass (DM) basis. Duncan's multiple range test and Pearson's correlation analysis were conducted using IBM SPSS Statistics 26, while the graphs were generated using Origin 8.0.

## 3 Results and discussion

### 3.1 Multi-dimensional microstructure changes of apple treated by US

Ultrasonic treatment significantly affected the hardness, chewability, springiness, and fracturability of apple slices (Table. S1). Specifically, after 10 min of ultrasonic treatment, the hardness and chewability of the apple slices decreased by 24.97 % and 41.89 %, respectively. The softening of fruit and vegetable tissues resulting from ultrasonic treatment can be attributed to acoustic cavitation, which causes the breakdown of the cell wall and reduces intercellular adhesion (Sila et al., 2010). Generally, the disruption of the cell wall is positively correlated with both processing time and ultrasound intensity (Wang, He & Wang et al., 2024).

**Fig. 1:**
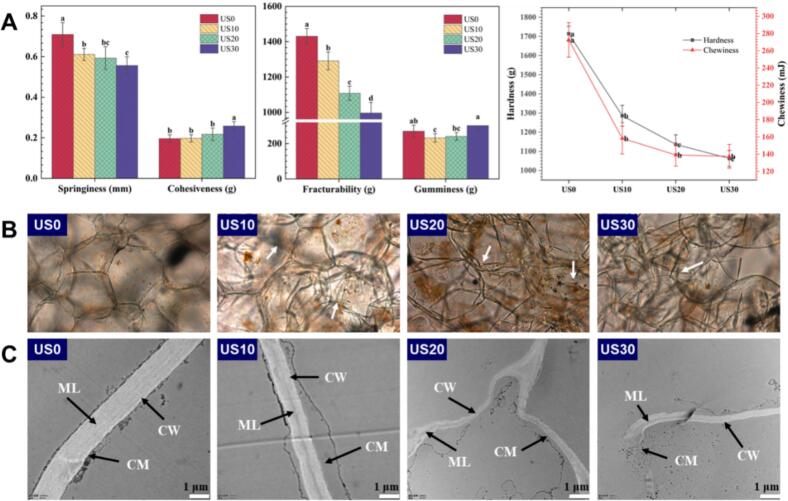
Microscopy images of fresh apple tissues with ultrasonic treatment. US: ultrasonic; CW: cell wall; CM: cell membrane; ML: middle layer of the cell walls.

The effects of different ultrasonic treatments on cell structure and cell wall ultrastructure are depicted in Fig. 1A & 1B, respectively. The micrographs reveal that the untreated sample exhibits a uniform cell wall thickness, with the cell membrane and attached organelles tightly bound to the cell wall. The cell outlines are clearly defined, and adjacent cells are closely fitted together. In plant cells, the middle lamella is situated between two adjacent cells, functioning as a shared membrane that binds the cells together and buffers compression between them, it is also referred to as the intercellular layer (Hua et al., 2023; Liu et al., 2023).

As the duration of ultrasonic (US) treatment was prolonged, the ultrastructure of the sample cell walls underwent significant alterations. Observations indicated that the space between adjacent cells widened, the middle lamella significantly dissolved, and the cell membrane along with its attachments gradually detached from the cell wall, compromising the integrity of the cell structure (Fig. 1B). In the samples treated with US20 and US30, microcracks and even fractures in the cell walls were observed, along with the detachment and dissolution of cell membranes and attachments, which dispersed within the cells, ultimately resulting in the destruction of the cell tissues. Comparable structural modifications, including cell wall disorganization and enhanced permeability, were consistently observed in mulberry (Wang, He & Wang et al., 2024), strawberry (Zhang et al., 2020), and cherry fruits (Zang et al., 2025) following ultrasound-assisted processing.

### 3.2 Changes in water status and cell-wall polysaccharide fractions of apple slices treated by US

**Fig. 2:**
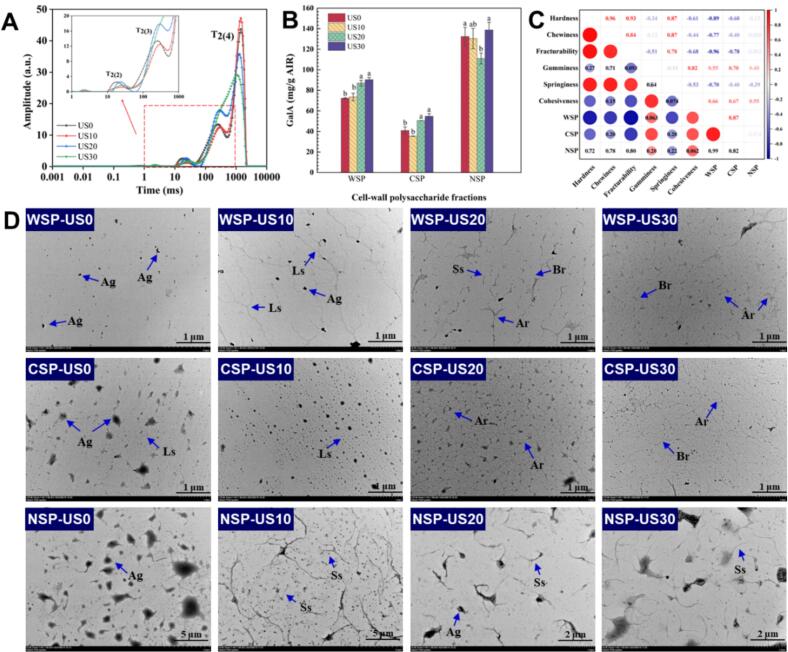
Effect of ultrasonic treatment on the water status, cell-wall polysaccharide fractions and ultrastructure of fresh apple slices. WSP: water soluble pectin; CSP: chelate-soluble pectin; NSP: sodium‑carbonate-soluble pectin; Ag: aggregation structure; Ar: arborization-like structure; Br: branched chain; Ls: long straight chain; Ss: short straight chain; AIR: alcohol-insoluble residue.

Nuclear magnetic resonance (NMR) transverse relaxation time (T_2_) is widely employed to examine the distribution and variations of water both within and between cells. Fig. 2A depicts the distribution of CPMG protons in apple slices subjected to various ultrasound treatments. The T_2_ curves of the apple slices exhibit three distinct peaks. The first peak, characterized by the shortest relaxation time (T_2(2)_ = 18.04 to 27.05 ms), corresponds to water that is tightly bound to cell wall macromolecules. The second peak (T_2(3)_ = 261.27 to 301.21 ms) represents cytoplasmic and intercellular water, while the third peak (T_2(4)_ = 1122.67 to 1431.46 ms) corresponds to free water, which is the most mobile form of water found in the vacuole.

In comparison to the control group (US0), the ultrasound-treated samples demonstrated a significant shift towards longer relaxation times, indicating that ultrasound treatment improved water mobility within the apple tissue, with this effect becoming more pronounced as treatment duration increased. In the US10 sample, the heights of the T_2(2)_ and T_2(3)_ peaks decreased, while the height of the T_2(4)_ peak increased significantly, suggesting that bound water and intracellular water were converted into free water. These results are primarily caused by the cavitation effects of ultrasound, which disrupt the cellular ultrastructure of cell walls and membranes (Fig. 1), thereby releasing bound water and transforming it into more freely mobile water. Additionally, the free water content increased in all groups subjected to 10 min of ultrasound treatment, aligning with the mass transfer results observed in the US10 sample.

Fig. 2B illustrates the effect of ultrasound treatment on the contents of water-soluble pectin (WSP), chelate-soluble pectin (CSP), and sodium‑carbonate-soluble pectin (NSP) in apple slices. Among the apple samples across all treatment groups, the content of NSP was consistently the highest, ranging from 110.99 ± 5.28 mg/g to 138.75 ± 7.35 mg/g AIR. This was followed by the WSP content (72.15 ± 0.55 mg/g to 90.35 ± 2.11 mg/g AIR), while the CSP content was the lowest (35.31 ± 0.26 mg/g to 54.69 ± 2.66 mg/g AIR). These results indicate that galacturonic acid (GalA) was predominantly concentrated in the NSP fraction. Ultrasound treatment facilitated the dissolution of pectin, as evidenced by a significant increase in the contents of WSP and CSP. This trend is consistent with the findings of Pieczywek et al. (2017) regarding the effects of ultrasonic treatment on the hardness and microstructure of apple cell walls. The increase in WSP content may be attributed to the depolymerization of pectin macromolecules induced by the cavitation and thermal effects of ultrasound treatment, resulting in the conversion of water-insoluble pectin into water-soluble pectin (Wang, He & Wang et al., 2024).

Fig. 2B indicates that the NSP content initially decreased before experiencing a slight increase with prolonged ultrasound treatment duration; however, this change was not statistically significant. This finding suggests that the impact of ultrasound treatment on NSP was minimal and that a longer treatment duration is necessary for any significant effects to become apparent. Ultrasound treatment enhanced the dissolution and depolymerization of pectin within the cell walls of apple slices, resulting in the disruption of covalent bonds in NSP and the subsequent loss of cell wall integrity (Xiao et al., 2025). This ultimately led to the softening of the fruit and vegetable tissue structure, consistent with the previously mentioned findings regarding structural changes.

The alterations in CSP and NSP content were closely linked to the softening of the fruit and vegetable tissue structure. Therefore, a correlation analysis was performed to examine the relationship between texture parameters and pectin content. Fig. 2C illustrates a significant negative correlation between hardness, chewy-ability, brittleness, and WSP content in apple slices, indicating that an increase in WSP content contributes to the softening of the tissue structure. Mierczyńska et al. (2015) reported that during the post-harvest storage of fruits and vegetables, enzymatic depolymerization resulted in an increase in pectin viscosity across all fractions, with the most pronounced effect observed in WSP. As a result, the increased dissolution of WSP following ultrasonic treatment reduces the viscosity of the cell wall space, enhances cell wall permeability, facilitates the transport of water and solutes, and improves water absorption capacity (Ni et al., 2023). Pectin acts as a plasticizer within the cell wall, interacting with the cellulose-hemicellulose network. Although cellulose and hemicellulose are more stable than pectin, their network may relax, leading to the softening of the tissue structure (Wang, He & Wang et al., 2024).

Fig. 2D illustrates the effects of ultrasound treatment on the structures of WSP, CSP, and NSP in apple slices. The non-ultrasonic sample displays numerous polymeric structures, whereas the pectin in the US10 sample begins to depolymerize, leading to the formation of several long, straight chains. As the duration of ultrasound treatment increases, the long straight chains break, resulting in an increased proportion of branched and dendritic structures. The CSP chains display a characteristic branched structure and form a network in conjunction with long chains, which are interconnected through ionic bonding and function as “bridges” (Ni et al., 2023; Xing et al., 2023). Ultrasound treatment resulted in significant degradation of the long chains in CSP. With extended treatment duration, the long straight chains degraded into shorter straight chain structures, leading to a gradual opening of the polymerization structure. The pectin network structure became denser, primarily due to dendritic connections. The proportion of branched chain structures increased in the US30 sample. The ultrastructure of NSP differs significantly from that of WSP and CSP, primarily displaying hair-like aggregations. Ultrasound treatment results in the noticeable dispersion of NSP aggregates, leading to the formation of uniformly dispersed short chain structures with reduced chain length. In conclusion, ultrasound treatment caused significant depolymerization and degradation of pectin within the cell wall, resulting in the disruption of the overall cell structure. This elucidates the mechanism by which ultrasound treatment softens the tissue of apple slices.

### 3.3 Drying characteristics

**Fig. 3:**
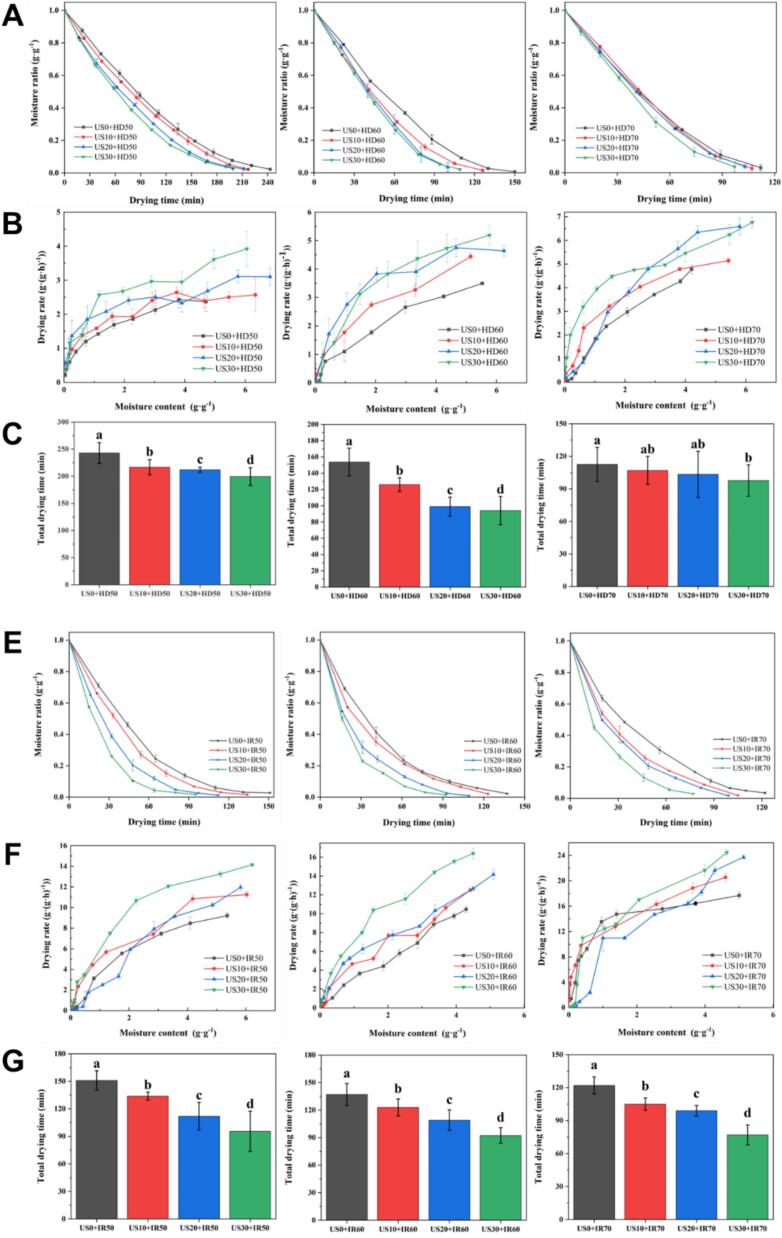
Effect of ultrasonic treatment on the drying kinetics and total drying time of apple slices. HD: hot-air drying; IR: Infrared drying.

Fig. 3 illustrates the variation of moisture ratio in apple slices with drying time using various US treatments and drying methods including HD and IR. As the drying time increases, the water content in the apple slices gradually decreases. For the same drying time, the water content in the ultrasonically treated apple slices is lower than that of the untreated samples. This is attributed to the ultrasonic pretreatment disrupting the cellular structure, particularly the intercellular layer, which creates additional pathways for moisture migration and enhances water mobility. Consequently, under identical drying conditions, moisture can be more efficiently transported to the surface and evaporated. At 50 °C, IR demonstrated significantly enhanced drying kinetics for apple slices compared to HD. This acceleration stems from resonant absorption of mid-infrared radiation by O—H vibrational modes in water molecules, generating internal vapor pressure gradients that amplify moisture transport. Conversely, at elevated temperatures (≥60 °C), thermal energy dominates the dehydration process, shifting the rate-limiting mechanism to surface evaporation as confirmed by converging effective diffusivity values (Chen et al., 2025).

The drying curves of apple slices after the combination of US and HD (Fig. 3A). At an HD temperature of 50 °C, the moisture content of apple slices decreased more rapidly and the drying time shortened with the increasing US time. As demonstrated in 3.1, 3.2, this phenomenon occurs because prolonged ultrasonic pretreatment disrupts cellular structural integrity, increasing the water-soluble pectin content. This increase in pectin promotes the conversion of bound water into free water, which migrates and evaporates more readily, thereby enhancing moisture transport and ultimately reducing drying time. As the drying temperature increased, the impact of US on the drying curve diminished. At a drying temperature of 60 °C, there was no notable disparity in the drying time between the apple slices treated with ultrasonic for 20 min and 30 min. This suggests that under these conditions, the impact of US for 20 min and 30 min on the drying process of apples was comparable. When the drying temperature reached 70 °C, the rate of moisture content reduction of apple slices in the first 20 min of the US group was not noticeably influenced by the ultrasonic time (Fig. 3B&C). However, the drying time of the ultrasonic 30 min treatment group was significantly reduced compared to the sample without ultrasonic treatment. This suggests that the drying effect of US on apple slices is diminished at higher drying temperatures, and it may require a longer US time to achieve a noticeable drying effect. In a study by Bai et al. (2022) it was found that when drying apple slices with hot air at 60 °C, a 10-min ultrasonic treatment did not notably affect the drying time, while prolonging the ultrasonic time to 30 min and 60 min resulted in a 16.7 % reduction in drying time. Thus, it was proposed that only an adequate US time could open the micro-pores of apple cells. This aligns with the findings of the present study, where the hot air temperature was 70 °C.

Fig. 3E depicts the moisture ratio curve of apple slices after US under infrared drying. The ultrasonic time has a significant impact on the moisture content change rate and drying time, and the trend is consistent across different temperatures (Fig. 3F&G). Corroborated by the data from Fig. 3A, ultrasonic treatment consistently reduced the drying time across all drying methods employed. It is noteworthy that while the moisture change rate and drying time of hot air-dried apple slices were predominantly influenced by temperature, the moisture content decline rate of infrared-dried slices increased markedly with extended ultrasonic treatment, consequently leading to a corresponding shortening of the drying time. The divergent effects of US on the drying of apple slices under the two drying methods may be attributed to their different drying principles. HD involves moist heat diffusion through convection, whereas IR drying relies on heat transfer through radiation. Temperature changes have distinct effects on the two heat transfer modes, leading to divergent drying effects on apple slices under the combined ultrasonic action. This is consistent with the observed trends in the microstructural changes under different treatments. For instance, the drying curves of apple slices dried by hot air at 70 °C showed minimal variation, accompanied by relatively slight microstructural alterations. This suggests that under high-temperature hot air-drying conditions, the sample surface is highly prone to rapid hardening, resulting in the formation of a dense layer that masks the benefits of ultrasonic pretreatment in disrupting cellular structures. Consequently, shorter ultrasonic treatment durations (10 and 20 min) proved ineffective. In contrast, infrared drying, leveraging its internal heating mechanism, effectively utilizes the microchannels created by ultrasound to vigorously facilitate moisture transfer through internal steam pressure differences, thereby demonstrating a more pronounced drying effect.

### 3.4 Color attributes

**Fig. 4:**
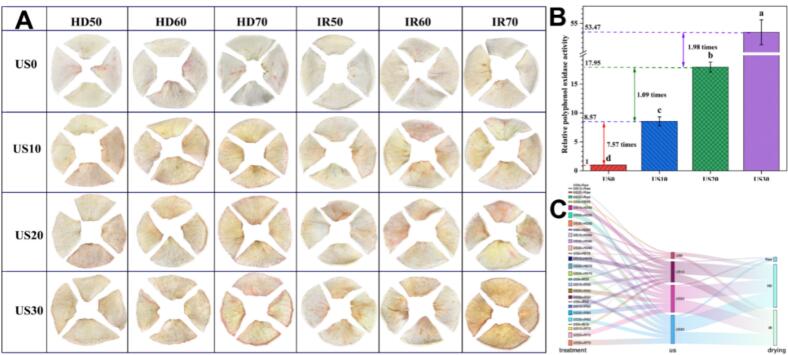
Effect of ultrasonic treatment on the color attributes of dried apple slices and activity of polyphenol oxidase time of fresh apple slices.

Color serves as a sensory indicator for consumers to directly observe the quality characteristics of dry apple slices. It is also an important parameter for evaluating their quality. Fig. 4 and Table S3 present the photographs and color parameters of dried apple slices subjected to different US and drying methods. Compared to the sample without US, the *L*^⁎^ value of the apple slices pretreated with US decreased significantly, while the *a*^⁎^ value increased significantly. As the ultrasonic time increased, the apple slices exhibited a color with enhanced redness. At a drying temperature of 70 °C, both hot air and infrared drying methods resulted in pink-colored apple slices, particularly in the groups treated with ultrasonic for 20 min and 30 min. Among these groups, the *a*^⁎^ value of US30 + IR70 was the highest, reaching 14.22, which was 5.21 times greater than that of the control groups. This color change may result from the cavitation effect and sono chemical action during the ultrasonic process, which increase intercellular spaces and micro-pores to enlarge the contact area between ultrasound waves and apple slices, thereby activating polyphenol oxidase (PPO) and increasing the occurrence of enzymatic browning reactions during drying (Zhang et al., 2022). However, no obvious redness was observed in the ultrasonic process of fresh apple slices. Therefore, it is likely that the combination of US with different drying methods and temperatures resulted in this color change. Previous research (Gonçalves et al., 2023) also found that US pretreatment had no significant effect on the color parameters of guava slices, whereas during the drying process, greater reductions in *L*^⁎^, *a*^⁎^, and *b*^⁎^ values were observed in pretreated samples. They attributed this phenomenon to prolonged exposure to elevated temperatures during drying, coupled with faster moisture outflow facilitated by the pretreatment during this process.

Polyphenol oxidase (PPO) is an oxidoreductase enzyme that utilizes oxygen to catalyze the hydroxylation of single phenols into o-diphenols. Subsequently, the generated o-diphenols are further oxidized into dark o-quinones, which then undergo non-enzymatic secondary reactions through condensation reactions to synthesize the brown pigment melanin (Arnold & Gramza-Michałowska, 2022). Fig. 4B demonstrates a significant increase in PPO enzyme activity with the application of US. The PPO activity increased gradually from one unit in the control group to 53.47. Specifically, for every 10 min of US time increase, the enzyme activity increased by 7.57 times, 1.09 times, and 1.98 times, respectively. This surge in PPO enzyme activity is highly consistent with the aforementioned change trend in color parameters (decrease in *L*^⁎^ value), demonstrating that ultrasonic treatment can significantly enhance PPO activity, leading to enzymatic browning and color darkening in apple slices. The application of ultrasound increased the activity of the PPO enzyme, which could be attributed to the dissociation of the PPO structure, leading to enhanced activity (Iqbal et al., 2019). The activity of the PPO enzyme is closely associated with the color development of apple slices. However, Bai et al. (2022) found that US reduced the *a*^⁎^ value of apples, which contradicts the conclusion of this study. This discrepancy may be attributed to variations in the raw materials' varieties and origins, as suggested by Arnold and Gramza-Michałowska (2022) in their review. Natural antioxidants in plants, such as vitamin C and phenolics, can alter enzyme activity and inhibit enzymatic browning. The effect on enzyme activity is influenced by the varieties and processing methods, as different varieties and phenolic components exhibit variations after processing. Certain phenols with lower molecular weight and smaller monomeric compounds (e.g., catechin, p-coumarinic acid, chlorogenic acid) in apples serve as more effective substrates for PPO (Serra et al., 2021). Serra et al. measured the PPO and POD activities of freshly cut samples from 14 Italian apple varieties. Granny Smith and Cripps Pink, which had the lowest content of coumarin and chlorogenic acid, exhibited lower PPO and POD activities within 24 h after cutting. Song et al. (2007) found a close relationship between non-enzymatic browning and epicatechin, catechin, chlorogenic acid, and caffeic acid. This explains why, in our study, despite the different changing trends in the content of chlorogenic acid and epicatechin, the color value still exhibited an increasing trend in the *a*^⁎^ value with the extension of ultrasonic time. This phenomenon is attributed to PPO enzymatic browning and polyphenol-mediated non-enzymatic browning.

### 3.5 Rehydration property

**Fig. 5:**
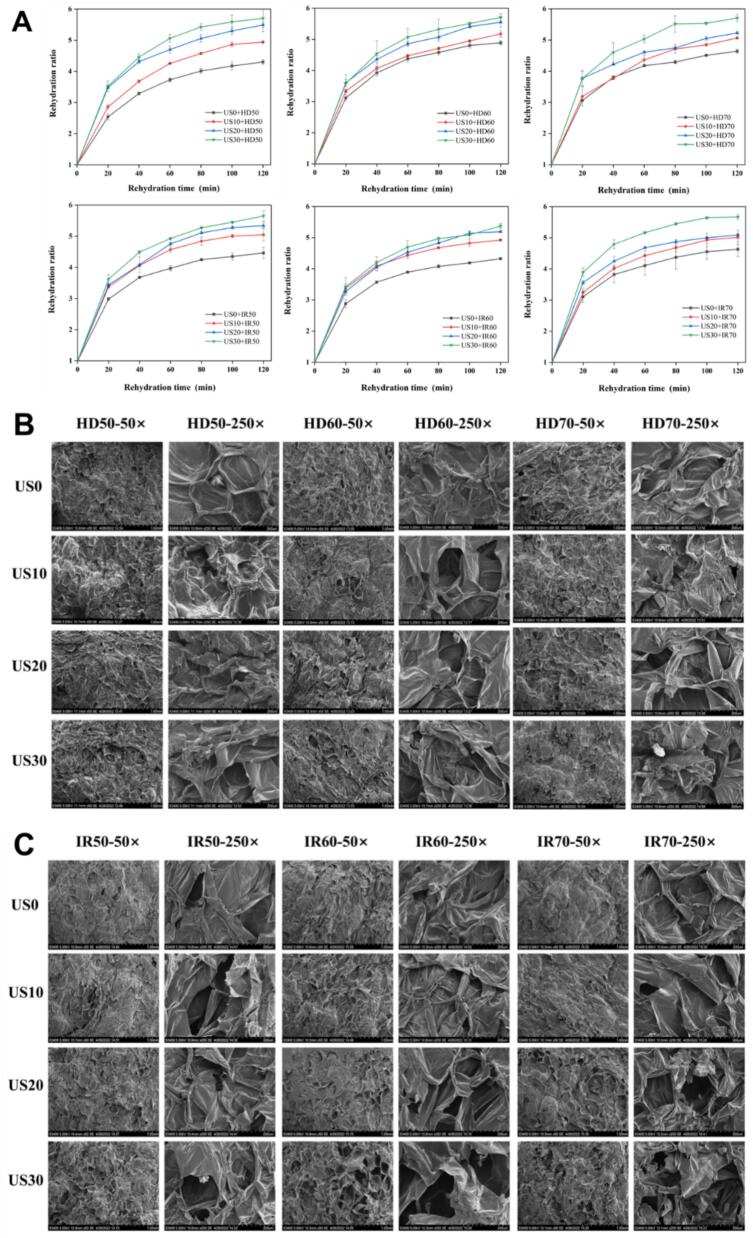
Effects of different ultrasonic pretreatments on rehydration dynamics curve and microstructure of HD apple slices and IR apple slices.

The rehydration rate is a crucial parameter for evaluating the structural looseness of dried products, representing the reverse process of drying. Fig. 5A illustrates the change in rehydration ratio of dried apple slices over time under various US and drying methods. As the rehydration time increased, the rehydration ratio of apple slices gradually increased. Under identical drying conditions, US facilitated the rehydration of dried apple slices. Moreover, as the ultrasonic time increased, the rehydration rate of apple slices also increased. This may be primarily because ultrasonic pretreatment disrupts the cellular structure and increases the content of water-soluble pectin, leading to enlarged intercellular spaces and the formation of additional microchannels. As a result, the dried apple slices develop a more porous structure, thereby significantly enhancing moisture penetration into the cells. The disparity in the impact of HD and IR on the rehydration ratio is somewhat associated with the drying curve under identical conditions. Observations reveal that at a hot air temperature of 70 °C, the rehydration rate of groups treated with ultrasound for 30 min is significantly higher than that of the first three groups. Conversely, at an infrared temperature of 60 °C, there is no significant difference in the rehydration rate of apple slices treated with ultrasound for 20 min and 30 min. These findings indicate that the rehydration performance of the dried product is directly influenced by the pretreatment and drying conditions, as well as their impact on the product structure, which aligns with previous studies on ultrasonic pretreatment applied to strawberries and lotus seeds, and different drying methods for yuba (Bi et al., 2024; Zhang, Wang, et al., 2024; Zhao et al., 2021). The extent of cellular and structural damage will alter the hydration capacity and rehydration degree of the samples (Zhang, Ding, et al., 2024).

The microstructure of the cross section of apple slices subjected to different pretreatments and drying methods is depicted in Fig. 5B and C Noticeable variations in the morphological characteristics of apple slices were observed among different treatment groups. Apple slices without ultrasound or those treated with short-time ultrasound at lower temperatures exhibited relatively flat surfaces and fewer gaps. As the ultrasonic time increased and the drying temperature rose, the surface tissue of apple slices experienced significant shrinkage, cell collapse, and the emergence of larger holes on the surface, as observed in samples such as US30 + HD60, US30 + HD70, US20 + IR70, US30 + IR70, and others. When subjected to identical pretreatment conditions and drying temperatures, the infrared-dried samples exhibited more pronounced collapse and a higher number of holes compared to the HD samples. This phenomenon may be attributed to the faster evaporation rate of water in IR compared to HD, which expedites the shrinkage and collapse of the tissue (Monteiro et al., 2020). The images captured after 20 and 30 min of US reveal evident cell collapse and shrinkage, along with the presence of numerous holes between tissues, irrespective of the drying method and temperature. This phenomenon can be attributed to the prolonged removal of air between cells, allowing a substantial influx of water into the intercellular space and increasing the contact area between water and cell tissue, thereby promoting a higher mass transfer area. Consequently, rapid water evaporation is facilitated during the drying process.

### 3.6 Major secondary metabolites and antioxidant activity in vitro

**Fig. 6:**
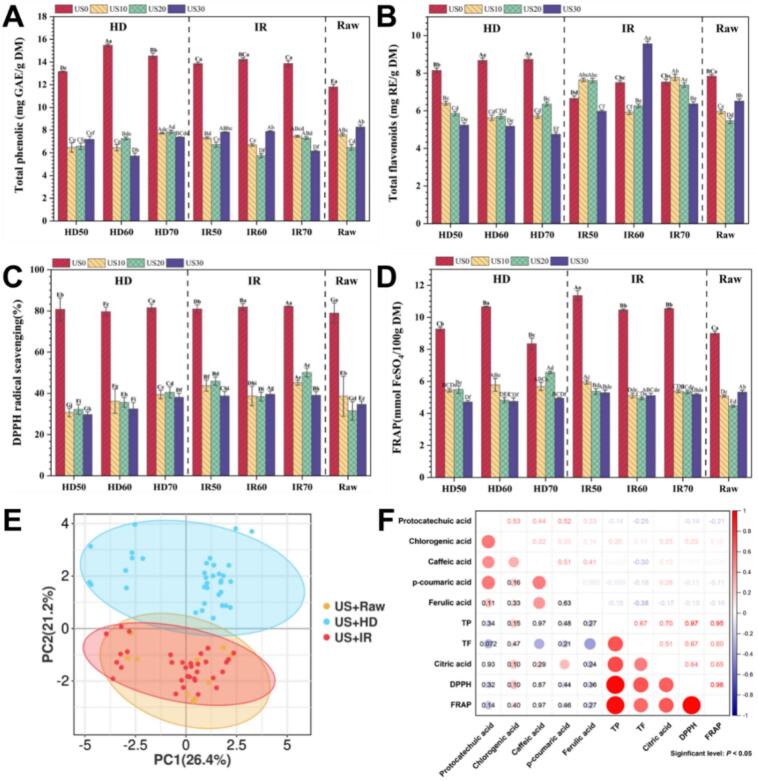
Effects of different ultrasonic pretreatments on major secondary metabolites and antioxidant activity in vitro of HD apple slices and IR apple slices.

Fig. 6A and B depict the contents of total phenols and flavonoids in dried apple slices, respectively. Compared to the group without US, the contents of total phenols and total flavonoids in ultrasonically treated apple slices exhibited a significant decrease, with no significant effect observed for ultrasonic time and drying temperature. This phenomenon may be attributed to ultrasound-induced disruption of cellular integrity, which not only enhances the leaching of water-soluble compounds during processing but also increases their exposure to polyphenol oxidase, thereby causing degradation. Similar findings were reported in the experimental study of Bozkir et al. (2019). Furthermore, the total phenol content of IR samples was significantly higher than that of HD samples at 50 °C, whereas it was significantly lower at 70 °C. The total flavonoid content of IR samples was significantly higher than that of HD samples at all three temperatures. These findings may be attributed to the differential thermal degradation kinetics and moisture migration patterns associated with distinct drying mechanisms, highlighting the method-dependent matrix interactions under identical pretreatment conditions.

The alterations in antioxidant activity of apple slices subjected to various pretreatments were assessed using DPPH and FRAP methods (Fig. 6C & 6D). The antioxidant capacity of dried apple slices pretreated with ultrasound exhibited a substantial decline compared to samples without ultrasound. Moreover, the antioxidant capacity slightly decreased with an increase in ultrasonic time, although the impact of ultrasonic time was not significant. Notably, the alteration trend of DPPH was in line with that of total flavonoids, while that of FRAP was consistent with total phenols. This direct correlation suggests that the antioxidant capacity reduction primarily stems from phenolic and flavonoid degradation through ultrasound-induced oxidative reactions. Kahraman et al. (2021) also reported a similar trend.

Phenolic compounds play a crucial role as nutrients and flavor components in apples. This study analyzed 10 different phenolic compounds in various dried apple slices, namely Gallic acid, protocatechuic acid, chlorogenic acid, caffeic acid, epicatechin, p-coumaric acid, ferulic acid, ellagic acid, phloridin, and quercetin. The specific concentrations are presented in Table 1. The main phenolic compounds identified were chlorogenic acid, epicatechin, and phloridin, with concentrations ranging from 54.66 to 141.53 mg/100 g DM, 12.45 to 44.64 mg/100 g DM, and 14.26 to 19.14 mg/g DM, respectively. According to Herranz et al. (2018), caffeic acid and chlorogenic acid were identified as the most significant phenolic compounds in Fuji apples, while other compounds like ferulic acid were also detected.

**Table 1:** Effects of different ultrasonic pretreatment and drying conditions on the content of phenolic compounds of apple slices.

Treatment	Phenolic compounds (mg/100 g DM)									
	Gallic acid	Protocatechuic acid	Chlorogenic acid	Caffeic acid	Epicatechin	p-coumaric acid	Ferulic acid	Ellagic acid	Phloridin	Quercetin
US0 + Raw	3.82 ± 0.01^c^	1.59 ± 0.13^b^	75.31 ± 1.60^a^	2.18 ± 0.05^ab^	37.59 ± 1.06^a^	6.23 ± 0.23^bc^	8.28 ± 1.12^a^	6.36 ± 0.80^ab^	18.30 ± 1.65^a^	16.11 ± 0.67^a^
US10 + Raw	4.41 ± 0.10^b^	1.77 ± 0.08^b^	59.8- ± 0.07^c^	2.59 ± 0.12^a^	23.88 ± 2.56^b^	6.04 ± 0.52^c^	6.73 ± 0.30^b^	6.34 ± 0.27^ab^	16.48 ± 2.54^ab^	15.07 ± 3.47^ab^
US20 + Raw	4.67 ± 0.03^a^	2.56 ± 0.26^a^	58.43 ± 1.15^c^	2.18 ± 0.24^ab^	7.87 ± 0.87^c^	5.80 ± 0.30^cd^	8.14 ± 0.36^ab^	6.61 ± 0.93^ab^	15.48 ± 2.18^ab^	12.92 ± 2.49^ab^
US30 + Raw	3.42 ± 0.06^d^	1.83 ± 0.07^b^	64.22 ± 1.21^b^	2.09 ± 0.10^b^	33.78 ± 1.26^a^	5.91 ± 0.28^cd^	6.79 ± 0.89^b^	6.62 ± 0.83^ab^	14.21 ± 0.19^b^	16.24 ± 3.17^a^
US0 + HD50	5.06 ± 0.01^ab^	3.02 ± 0.31^ab^	141.53 ± 2.47^d^	2.28 ± 0.23^bcd^	15.43 ± 2.71^ab^	6.20 ± 0.33^bc^	8.06 ± 2.13^a^	7.08 ± 0.11^a^	16.54 ± 0.60^ab^	11.59 ± 2.16^ab^
US10 + HD50	5.15 ± 0.45^ab^	2.93 ± 0.78^ab^	140.64 ± 2.66^cd^	2.26 ± 0.41^bcd^	16.38 ± 1.73^ab^	6.47 ± 0.32^abc^	8.16 ± 2.01^a^	7.15 ± 0.23^a^	18.38 ± 1.76^ab^	13.88 ± 1.14^ab^
US20 + HD50	5.68 ± 0.72^ab^	2.98 ± 0.25^ab^	124.15 ± 3.66^cd^	2.54 ± 0.12^ab^	12.45 ± 0.76^b^	6.42 ± 0.06^abc^	9.14 ± 1.20^a^	7.60 ± 0.76^a^	16.53 ± 0.24^ab^	11.06 ± 2.84^ab^
US30 + HD50	5.70 ± 1.03^ab^	2.78 ± 0.78^b^	108.91 ± 7.94^cd^	2.30 ± 0.14^bcd^	15.45 ± 3.24^ab^	6.76 ± 0.47^abc^	7.20 ± 1.32^ab^	7.48 ± 0.20^a^	18.59 ± 1.42^ab^	14.42 ± 2.54^a^
US0 + HD60	3.80 ± 0.79^b^	2.92 ± 0.38^ab^	140.46 ± 6.23^bc^	2.43 ± 0.32^abcd^	17.48 ± 2.36^a^	6.83 ± 0.42^ab^	6.93 ± 1.06^b^	6.79 ± 0.48^a^	16.70 ± 1.77^ab^	11.20 ± 3.13^ab^
US10 + HD60	6.90 ± 0.94^a^	2.95 ± 0.27^ab^	114.66 ± 2.21^abc^	2.49 ± 0.02^abc^	17.08 ± 1.14^ab^	6.32 ± 0.24^abc^	8.70 ± 0.62^a^	7.00 ± 0.44^a^	17.91 ± 0.74^ab^	10.51 ± 2.27^ab^
US20 + HD60	5.08 ± 0.18^ab^	3.07 ± 0.06^ab^	124.86 ± 1.13^abc^	2.08 ± 0.25^cd^	16.37 ± 2.65^ab^	6.67 ± 0.59^abc^	7.18 ± 1.84^ab^	6.92 ± 1.24^a^	16.86 ± 0.96^ab^	13.59 ± 0.83^ab^
US30 + HD60	7.29 ± 3.32^a^	3.90 ± 0.09^a^	111.22 ± 10.40^abc^	2.85 ± 0.16^a^	17.66 ± 2.73^a^	7.56 ± 0.18^a^	8.86 ± 0.67^a^	6.58 ± 1.38^ab^	16.54 ± 1.70^ab^	10.39 ± 1.83^b^
US0 + HD70	7.35 ± 0.32^a^	3.32 ± 0.02^ab^	145.05 ± 5.03^ab^	2.20 ± 0.29^bcd^	16.04 ± 2.20^ab^	6.14 ± 0.29^bc^	9.00 ± 0.55^a^	6.30 ± 0.45^ab^	19.14 ± 1.69^a^	13.01 ± 0.28^ab^
US10 + HD70	7.15 ± 1.30^a^	2.53 ± 0.65^b^	118.68 ± 2.03^ab^	2.36 ± 0.09^bcd^	13.11 ± 0.36^ab^	5.53 ± 0.67^c^	9.40 ± 0.50^a^	6.67 ± 0.05^ab^	16.86 ± 3.02^ab^	12.34 ± 1.37^ab^
US20 + HD70	6.51 ± 0.88^a^	3.22 ± 0.63^ab^	130.29 ± 12.58^ab^	2.02 ± 0.25^c^	12.58 ± 1.25^b^	5.93 ± 0.48^bc^	9.23 ± 1.06^a^	4.54 ± 0.44^b^	16.53 ± 1.61^ab^	13.55 ± 0.67^ab^
US30 + HD70	5.74 ± 1.24^ab^	2.87 ± 0.12^b^	93.25 ± 3.62^a^	2.36 ± 0.14^bcd^	13.88 ± 0.35^ab^	5.68 ± 0.66^bc^	9.22 ± 0.67^a^	6.73 ± 0.59^ab^	15.99 ± 0.65^b^	12.05 ± 2.55^ab^
US0 + IR50	3.29 ± 0.60^d^	2.07 ± 0.15^b^	65.77 ± 3.69^c^	2.12 ± 0.02^bc^	35.35 ± 0.48^ab^	5.93 ± 0.19^cd^	8.34 ± 0.71^ab^	7.45 ± 1.91^abc^	16.38 ± 0.31^ab^	14.63 ± 2.15^abc^
US10 + IR50	5.10 ± 0.18^abc^	1.70 ± 0.07^b^	77.32 ± 3.56^b^	2.04 ± 0.07^bc^	38.89 ± 1.37^ab^	6.96 ± 0.32^b^	7.00 ± 0.25^b^	9.88 ± 1.20^a^	16.23 ± 0.39^ab^	12.31 ± 0.48^bcd^
US20 + IR50	3.74 ± 0.88^cd^	1.92 ± 0.13^b^	75.89 ± 1.45^b^	2.06 ± 0.13^bc^	32.25 ± 0.39^bcd^	6.16 ± 0.95^cd^	7.39 ± 0.11^ab^	6.40 ± 1.38^bc^	16.36 ± 0.89^ab^	15.44 ± 1.96^ab^
US30 + IR50	4.88 ± 0.21^bcd^	1.67 ± 0.47^b^	54.66 ± 2.77^d^	2.29 ± 0.21^ab^	38.50 ± 6.41^ab^	5.96 ± 0.54^cd^	9.30 ± 0.17^a^	6.34 ± 0.51^c^	15.35 ± 0.54^bc^	17.76 ± 0.76^a^
US0 + IR60	5.40 ± 0.18^abc^	2.25 ± 0.72^b^	92.66 ± 3.31^a^	2.28 ± 0.26^ab^	36.40 ± 3.09^ab^	6.38 ± 0.30^bc^	8.15 ± 0.95^ab^	9.77 ± 0.44^ab^	16.41 ± 0.11^ab^	16.11 ± 1.22^a^
US10 + IR60	5.88 ± 0.68^ab^	4.38 ± 0.20^a^	77.94 ± 2.39^b^	2.48 ± 0.34^a^	44.64 ± 3.35^a^	9.79 ± 0.30^a^	9.18 ± 0.54^a^	7.38 ± 0.99^abc^	17.77 ± 0.82^a^	14.42 ± 1.34^abc^
US20 + IR60	6.57 ± 0.64^ab^	2.23 ± 0.58^b^	74.84 ± 0.76^b^	1.94 ± 0.05^bc^	24.89 ± 1.42^cd^	5.87 ± 0.18^cd^	8.03 ± 0.47^ab^	5.09 ± 0.30^c^	15.22 ± 0.21^bc^	10.11 ± 0.20^d^
US30 + IR60	4.84 ± 0.06^bcd^	1.80 ± 0.25^b^	91.95 ± 1.54^a^	2.00 ± 0.16^bc^	34.99 ± 3.08^ab^	5.85 ± 0.13^cd^	6.54 ± 0.30^b^	5.85 ± 0.06^c^	14.91 ± 0.15^bc^	10.26 ± 0.63^d^
US0 + IR70	6.71 ± 0.19^a^	0.21 ± 0.03^c^	79.08 ± 1.15^b^	2.14 ± 0.13^bc^	32.99 ± 1.21^bc^	5.87 ± 0.20^cd^	7.59 ± 0.45^ab^	6.21 ± 0.19^c^	15.15 ± 0.02^bc^	11.15 ± 0.87^cd^
US10 + IR70	5.47 ± 0.74^abc^	2.40 ± 0.36^b^	66.57 ± 0.87^c^	1.87 ± 0.14^c^	22.80 ± 1.18^d^	5.65 ± 0.20^cd^	7.71 ± 0.43^ab^	6.31 ± 0.68^c^	15.71 ± 0.93^bc^	9.85 ± 0.54^d^
US20 + IR70	5.45 ± 0.80^abc^	1.65 ± 0.03^b^	88.77 ± 2.25^a^	1.97 ± 0.18^bc^	32.34 ± 2.99^bcd^	6.02 ± 0.19^cd^	7.58 ± 0.90^ab^	7.56 ± 1.38^abc^	14.42 ± 0.30^c^	11.24 ± 1.22^cd^
US30 + IR70	5.28 ± 0.63^abc^	1.62 ± 0.02^b^	65.20 ± 0.40^c^	2.07 ± 0.10^bc^	24.69 ± 2.23^cd^	5.57 ± 0.09^d^	7.92 ± 0.62^ab^	4.59 ± 0.05^c^	14.26 ± 0.09^c^	10.21 ± 0.18^d^

Note: Different superscript letters in the same column reveal that there are significant differences under the same treatment (*P* < 0.05).

For hot-air dried (HD) apple slices, as the ultrasonic time increased, the epicatechin content increased, albeit not significantly (Table 1). However, it decreased slightly when the drying temperature was 50 °C and 60 °C, reaching 70 °C. For infrared-dried (IR) apple slices, US led to a non-significant increase in epicatechin content, while the chlorogenic acid content increased significantly (Table 1). The reduced loss of these compounds may be explained by ultrasound-induced modifications in cellular permeability that selectively retain specific phenolics. Furthermore, the chlorogenic acid content in infrared-dried apple slices was significantly lower than that in hot-air dried samples, while the epicatechin content was significantly higher in the infrared-dried samples compared to the hot-air dried ones. This divergence likely arises from infrared radiation's unique energy transfer mechanism that differentially affects phenolic stability through localized heating effects. Additionally, the effects of different temperatures on the phenolic compound contents in dried apple slices were examined under the same US and drying methods. The analysis revealed that temperature had varying effects on different phenolic compounds. For instance, the increase in drying temperature led to a rise in Gallic acid content in dried apple slices, potentially due to thermal liberation of bound forms, whereas the caffeic acid content generally decreased with increasing drying temperature owing to thermal lability of its ester bonds.

In this case, try to explain the following differences in the changes of different phenols. On the one hand, phenolic compounds may have some derivative reactions. For example, caffeic acid can be converted to caffeoylquinic acid under air drying (Lin et al., 2022), while Maillard reaction may produce some new phenols (Aydin & Gocmen, 2015). On the other hand, due to the effect of pretreatment on cell structure, the affinity between fruit cell wall and binding phenols may be weakened (Tao et al., 2021). In addition, the difference of phenolic content was caused by the difference of loss in the process of pretreatment, drying and phenolic extraction, such as the effect of enzyme activity change on phenols in ultrasonic process and the thermal degradation of phenols in drying process. These physical and chemical phenomena can lead to the increase or decrease of phenolic content. Zhu et al. (2022) showed that airborne US had a negative effect on the stability of some phenolic compounds in apple samples under air drying. Some studies have also found that the total phenol content of Sanhua plum, persimmon and cranberry dried by ultrasound-assisted air drying is lower than that of non-ultrasonic dried samples (Bozkir et al., 2019; Li et al., 2021; Nowacka et al., 2018). Critically, ultrasound exerts a dual effect by simultaneously enhancing mass transfer and generating reactive oxygen species, thereby establishing antagonistic pathways that govern compound-specific degradation kinetics (Nowacka et al., 2018).

### 3.7 Organic acids

The original data on the individual organic acid content of apple slices subjected to various drying treatments are presented in Table 2, and the original map of organic acids detected by HPLC in apples treated with different treatmentsare presented in Fig. S1. Ultrasonic pretreatment (US) increased the malic acid content in apple slices. As the duration of ultrasound treatment was extended, the malic acid content in apple slices initially increased and then decreased across the different drying methods. The loss of malic acid during the processing stages first decreased and then increased. Specifically, the loss rate of malic acid was lowest when the ultrasound duration was 10 min, while it was highest for the 30-min ultrasound treatment. This trend is likely due to relatively mild cellular disruption at shorter ultrasonic durations, which helps retain malic acid within the cells. In contrast, prolonged pretreatment for 30 min induces cell wall fracture and severe loss of cellular integrity (Fig. 1), potentially leading to malic acid leakage or degradation. The concentrations of malic acid, citric acid, oxalic acid and fumaric acid in the dried apple slices ranged from 12.43 to 26.32 mg/g DM, 0.18–11.24 mg/g DM, 0.84–3.36 mg/g DM and 0.19–0.37 mg/g DM (Table 2), respectively. The results indicate that the different drying temperatures and methods had negligible effects on the detected organic acids. Across all drying treatments, the malic acid content was significantly higher than the other organic acids, likely due to the high thermal stability of malic acid.

**Table 2:** Effects of different ultrasonic pretreatment and drying conditions on the content of organic acid of apple slices.

Treatment	Organic acid (mg/g DM)			
	Oxalic acid	Citric acid	Malic acid	Fumaric acid
US0 + Raw	2.96 ± 0.02^b^	2.74 ± 0.22^ab^	18.47 ± 0.02^a^	0.31 ± 0.01^a^
US10 + Raw	3.26 ± 0.05^a^	2.15 ± 0.17^b^	24.07 ± 0.41^b^	0.26 ± 0.00^b^
US20 + Raw	1.05 ± 0.03^c^	0.61 ± 0.06^c^	21.99 ± 0.38^c^	0.25 ± 0.01^b^
US30 + Raw	1.00 ± 0.00^c^	3.10 ± 0.16^a^	15.23 ± 0.52^a^	0.23 ± 0.01^c^
US0 + HD50	3.06 ± 0.02^a^	6.28 ± 0.17^b^	19.02 ± 1.27^abc^	0.32 ± 0.01^ab^
US10 + HD50	1.47 ± 0.70^bc^	0.53 ± 0.01^g^	20.19 ± 0.65^ab^	0.27 ± 0.03^bcd^
US20 + HD50	0.90 ± 0.03^c^	3.33 ± 0.41^d^	18.67 ± 1.93^bc^	0.26 ± 0.03^bcd^
US30 + HD50	0.90 ± 0.02^c^	1.42 ± 0.05^f^	19.44 ± 0.50^abc^	0.23 ± 0.01^d^
US0 + HD60	2.46 ± 0.09^ab^	11.24 ± 0.27^a^	21.93 ± 1.91^a^	0.33 ± 0.00^a^
US10 + HD60	1.06 ± 0.01^c^	2.45 ± 0.21^e^	18.93 ± 0.60^abc^	0.27 ± 0.04^abcd^
US20 + HD60	2.76 ± 1.03^a^	4.84 ± 0.23^c^	18.36 ± 0.32^bc^	0.25 ± 0.01^d^
US30 + HD60	0.98 ± 0.03^c^	1.44 ± 0.31^f^	12.50 ± 0.83^e^	0.25 ± 0.02^cd^
US0 + HD70	2.49 ± 0.55^ab^	4.67 ± 0.18^c^	14.13 ± 0.46^de^	0.31 ± 0.01^abc^
US10 + HD70	1.09 ± 0.03^c^	1.87 ± 0.04^ef^	19.40 ± 0.68^abc^	0.24 ± 0.01^d^
US20 + HD70	1.22 ± 0.05^c^	1.32 ± 0.10^fg^	21.04 ± 1.15^ab^	0.26 ± 0.01^cd^
US30 + HD70	1.00 ± 0.04^c^	1.63 ± 0.18^ef^	16.69 ± 0.18^cd^	0.24 ± 0.01^d^
US0 + IR50	1.69 ± 0.51^abc^	3.96 ± 0.76^c^	14.41 ± 1.42^de^	0.47 ± 0.07^a^
US10 + IR50	2.01 ± 0.10^bc^	3.53 ± 0.28^c^	21.44 ± 0.76^b^	0.31 ± 0.07^bc^
US20 + IR50	1.52 ± 0.76^abc^	0.98 ± 0.51^c^	15.98 ± 0.18^de^	0.29 ± 0.04^bc^
US30 + IR50	0.87 ± 0.02^c^	3.77 ± 0.70^c^	17.26 ± 0.53^cd^	0.26 ± 0.02^bc^
US0 + IR60	0.98 ± 0.05^bc^	6.49 ± 0.37^ab^	21.45 ± 0.51^b^	0.23 ± 0.20^bc^
US10 + IR60	1.53 ± 0.78^abc^	7.36 ± 7.27^abc^	20.08 ± 2.54^bc^	0.26 ± 0.06^bc^
US20 + IR60	0.84 ± 0.08^c^	0.18 ± 0.32^c^	12.43 ± 0.65^e^	0.26 ± 0.01^bc^
US30 + IR60	1.57 ± 0.08^abc^	2.66 ± 0.05^c^	12.81 ± 0.35^e^	0.30 ± 0.02^bc^
US0 + IR70	1.40 ± 0.06^abc^	6.02 ± 0.42^bc^	16.93 ± 0.39^cd^	0.37 ± 0.02^ab^
US10 + IR70	2.38 ± 0.12^ab^	1.50 ± 1.65^a^	17.27 ± 0.26^cd^	0.19 ± 0.16^c^
US20 + IR70	3.36 ± 0.04^a^	ND	26.32 ± 2.44^a^	0.26 ± 0.02^bc^
US30 + IR70	1.16 ± 0.04^bc^	1.63 ± 0.94^c^	16.59 ± 1.43^cd^	0.37 ± 0.03^ab^

Note: Different superscript letters in the same column reveal that there are significant differences under the same treatment (*P* < 0.05).

## 4 Conclusion

This study systematically elucidated the mechanism by which US pretreatment affects moisture migration and quality characteristics during the drying process of apple slices, utilizing a multi-angle microscopic structural observation system. US treatment significantly enhances the mass transfer rate by promoting the depolymerization of pectin in the cell wall and disrupting the microstructure, resulting in reductions in drying times for US-HD and US-IR apple slices of 13.31 % to 38.82 % and 32.60 % to 36.88 %, respectively. However, US treatment results in a reduction of total phenols and total flavonoids, thereby weakening antioxidant activity. Optimizing the pretreatment time is necessary to activate polyphenol oxidase (PPO) enzymes and improve color, thereby balancing quality and efficiency. Nevertheless, the surface pore structure induced by US enhances rehydration capacity and partially mitigates the negative effects of drying on nutritional quality by reducing the loss of key components, such as chlorogenic acid and malic acid. Overall, US pretreatment positively impacts the drying rate and quality of apple slices.

## CRediT authorship contribution statement

**Cheng Zhang:** Writing – original draft, Methodology, Investigation, Funding acquisition, Conceptualization. **Zina Lin:** Software, Methodology, Investigation, Data curation. **Xiaolong Li:** Software, Methodology, Investigation. **Jiachi Duan:** Methodology, Investigation, Formal analysis. **Jiaqi Liu:** Methodology, Investigation. **Shuang Luo:** Methodology, Investigation. **Liangyu Dai:** Software, Resources. **Bing Tian:** Resources, Project administration, Funding acquisition. **Jun Wang:** Writing – review & editing, Conceptualization. **Jun Li:** Writing – review & editing, Conceptualization.

## Declaration of competing interest

The authors declare that they have no known competing financial interests or personal relationships that could have appeared to influence the work reported in this paper.

## Data Availability

Data will be made available on request.

## Appendix A Supplementary data

Supplementary material Image 1

## References

[bb0005] Arnold M., Gramza-Michałowska A. (2022). Enzymatic browning in apple products and its inhibition treatments: A comprehensive review. Comprehensive Reviews in Food Science and Food Safety.

[bb0010] Aydin E., Gocmen D. (2015). The influences of drying method and metabisulfite pre-treatment on the color, functional properties and phenolic acids contents and bioaccessibility of pumpkin flour. LWT - Food Science and Technology.

[bb0015] Azoubel P.M., Baima M.D.M., Amorim M.D., Oliveira S.S.B. (2010). Effect of ultrasound on banana cv Pacovan drying kinetics. Journal of Food Engineering.

[bb0020] Bai J.W., Zhang L., Aheto J.H., Cai J.R., Wang Y.C., Sun L., Tian X.Y. (2022). Effects of different pretreatment methods on drying kinetics, three-dimensional deformation, quality characteristics and microstructure of dried apple slices. Innovative Food Science & Emerging Technologies.

[bb0025] Bao G., Tian Y., Wang K., Chang Z., Jiang Y., Wang J. (2024). Mechanistic understanding of the improved drying characteristics and quality attributes of lily (Lilium lancifolium Thunb.) by modified microstructure after pulsed electric field (PEF) pretreatment. Food Research International.

[bb0030] Ben-Arie R., Kislev N. (1979). Ultrastructural changes in the cell walls of ripening apple and pear fruit. Plant Physiology.

[bb0035] Benhamza A., Boubekri A., Atia A., Ferouali H.E., Hadibi T., Arıcı M., Abdenouri N. (2021). Multi-objective design optimization of solar air heater for food drying based on energy, exergy and improvement potential. Renewable Energy.

[bb0040] Bi J., Zhang J., Chen Z., Li Y., Obadi M., Liu W., Qin R., Zhang L., He H. (2024). Quality variation analysis and rehydration kinetics modeling of yuba subjecting to three different drying process. Food Chemistry: X.

[bb0045] Bozkir H., Ergün A.R., Serdar E., Metin G., Baysal T. (2019). Influence of ultrasound and osmotic dehydration pretreatments on drying and quality properties of persimmon fruit. Ultrasonics Sonochemistry.

[bb0050] Chen P.X., Wang Y.K., Zhu W.X., Wang X.W., Xing Y.J., Zhang T.T., Lv J.L. (2025). Effect of different drying techniques on moisture migration, nutritional profile and sensory attributes of peanut pods. LWT.

[bb0055] Chen Y., Li M., Dharmasiri T.S.K., Song X., Liu F., Wang X. (2020). Novel ultrasonic-assisted vacuum drying technique for dehydrating garlic slices and predicting the quality properties by low field nuclear magnetic resonance. Food Chemistry.

[bb0060] Chojnacka K., Mikula K., Izydorczyk G., Skrzypczak D., Witek-Krowiak A., Moustakas K., Kulazynski M. (2021). Improvements in drying technologies - efficient solutions for cleaner production with higher energy efficiency and reduced emission. Journal of Cleaner Production.

[bb0065] Corigliano O., Algieri A. (2024). A comprehensive investigation on energy consumptions, impacts, and challenges of the food industry. Energy Conversion and Management: X.

[bb0070] Gonçalves D.J.R., Costa N.D.A., Paiva M.J.D.A., Oliveira V.C.D., Maia N.M.A., Magalhães I.S., Júnior B.R.D.C.L.J. (2023). Ultrasonic pre-treatment to enhance drying of potentially probiotic guava (*Psidium guajava*): Impact on drying kinetics, Lacticaseibacillus rhamnosus GG viability, and functional quality. Food Research International.

[bb0075] Gowd V., Xu Y., Zhao J., Bao T., Xie J., Liang W., Chen W. (2016). Systematic study on phytochemicals and antioxidant activity of some new and common mulberry cultivars in China. Journal of Functional Foods.

[bb0080] Herranz B., Fernández-Jalao I., Alvarez M.D., Quiles A., Sánchez-Moreno C., Hernando I., de Ancos B. (2018). Phenolic compounds, microstructure and viscosity of onion and apple products subjected to in vitro gastrointestinal digestion. Innovative Food Science and Emerging Technologies.

[bb0085] Hua X., Li T., Wu C., Zhou D., Fan G., Li X., Cong K., Yan Z., Cheng X. (2023). Pulsed light improved the shelf life of apricot (after simulated long-distance air transportation) by regulating cell wall metabolism. Postharvest Biology and Technology.

[bb0090] Iqbal A., Murtaza A., Hu W., Ahmad I., Ahmed A., Xu X.Y. (2019). Activation and inactivation mechanisms of polyphenol oxidase during thermal and non-thermal methods of food processing. Food and Bioproducts Processing.

[bb0095] Kahraman O., Malvandi A., Vargas L., Feng H. (2021). Drying characteristics and quality attributes of apple slices dried by a non-thermal ultrasonic contact drying method. Ultrasonics Sonochemistry.

[bb0100] Kossah R., Nsabimana C., Zhao J., Chen H., Tian F., Zhang H., Chen W. (2009). Comparative study on the chemical composition of Syrian sumac (Rhus coriaria L.) and Chinese sumac (Rhus typhina L.) fruits. Pakistan Journal of Nutrition.

[bb0105] Krystian M., Ukasz W., Barba F.J., Sylwia S., Lorenzo J.M., Alessandro Z., Sara S. (2018). Enzymatic, physicochemical, nutritional and phytochemical profile changes of apple (Golden delicious L.) juice under supercritical carbon dioxide and long-term cold storage. Food Chemistry.

[bb0110] Li D.T., Deng L.Z., Dai T.T., Chen M.S., Liang R.H., Liu W., Sun J. (2022). Ripening induced degradation of pectin and cellulose affects the far infrared drying kinetics of mangoes. Carbohydrate Polymers.

[bb0115] Li L., Yu Y., Xu Y., Wu J., Yu Y., Peng J., An K., Zou B., Yang W. (2021). Effect of ultrasound-assisted osmotic dehydration pretreatment on the drying characteristics and quality properties of Sanhua plum (Prunus salicina L.). LWT.

[bb0120] Li X., Wu X., Bi J., Liu X., Guo C. (2019). Polyphenols accumulation effects on surface color variation in apple slices hot air drying process. LWT- Food Science and Technology.

[bb0125] Lin Z., Geng Z., Liang W., Zhu H., Ye J., Wang J., Xu H. (2022). Steam blanching and ethanol pretreatment enhance drying rates and improve the quality attributes of apple slices via microstructure modification. Journal of Food Processing and Preservation.

[bb0130] Liu M., Li G., Sun W., Li H., Fu J., Zong W., Han W. (2023). Effect of ultrasonic treatment on water-soluble pectin and degrading enzymes in cell wall of persimmon fruit during storage. Journal of Food Composition and Analysis.

[bb0135] Llavata B., Quiles A., Rosselló C., Cárcel J.A. (2025). Enhancing ultrasonic-assisted drying of low-porosity products through pulsed electric field (PEF) pretreatment: The case of butternut squash. Ultrasonics Sonochemistry.

[bb0140] Maskan M. (2001). Drying, shrinkage and rehydration characteristics of kiwifruits during hot air and microwave drying. Journal of Food Engineering.

[bb0145] Mierczyńska J., Cybulska J., Sołowiej B., Zdunek A. (2015). Effect of Ca^2+^, Fe^2+^ and Mg^2+^ on rheological properties of new food matrix made of modified cell wall polysaccharides from apple. Carbohydrate Polymers.

[bb0150] Monteiro R.L., Moraes J., Domingos J.D., Carciofi B., Laurindo J.B. (2020). Evolution of the physicochemical properties of oil-free sweet potato chips during microwave vacuum drying. Innovative Food Science and Emerging Technologies.

[bb0155] Ni B.J., Zielinska M., Wang J., Fang M.X., Sutar P.P., Li S.B., Xiao H.W. (2023). Post-harvest ripening affects drying behavior, antioxidant capacity and flavor release of peach via alteration of cell wall polysaccharides content and nanostructures, water distribution and status. Food Research International.

[bb0160] Nowacka M., Fijalkowska A., Wiktor A., Dadan M., Tylewicz U., Rosa M.D., Witrowa-Rajchert D. (2018). Influence of power ultrasound on the main quality properties and cell viability of osmotic dehydrated cranberries. Ultrasonics.

[bb0165] Önal B., Adiletta G., Crescitelli A., Matteo M.D., Russo P. (2019). Optimization of hot air drying temperature combined with pre-treatment to improve physico-chemical and nutritional quality of ‘Annurca’ apple. Food and Bioproducts Processing.

[bib301] Pieczywek P.M., Koziol A., Konopacka D., Cybulska J., Zdunek A. (2017). Changes in cell wall stiffness and microstructure in ultrasonically treated apple. Journal of food engineering.

[bb0170] Serra S., Anthony B., Sesillo F.B., Masia A., Musacchi S. (2021). Determination of post-harvest biochemical composition, enzymatic activities, and oxidative browning in 14 apple cultivars. Foods.

[bb0175] Sethi S., Joshi A., Arora B., Bhowmik A., Kumar P. (2020). Significance of FRAP, DPPH, and CUPRAC assays for antioxidant activity determination in apple fruit extracts. European Food Research and Technology.

[bb0180] Sila D.N., Van Buggenhout S., Duvetter T., Fraeye I., De Roeck A., Van Loey A., Hendrickx M. (2010). Pectins in processed fruits and vegetables: part II-structure function relationships. Comprehensive Reviews in Food Science and Food Safety.

[bb0185] Song Y., Zhai H., Liu J.B., Du Y.P., Chen F., Wei S.W. (2007). Polyphenolic compound and degree of browning in processing apple varieties. Scientia Agricultura Sinica.

[bb0190] Sun M., Zhuang Y., Gu Y., Zhang G., Fan X., Ding Y. (2024). A comprehensive review of the application of ultrasonication in the production and processing of edible mushrooms: Drying, extraction of bioactive compounds, and post-harvest preservation. Ultrasonics Sonochemistry.

[bb0195] Tao Y., Li D., Chai W.S., Show P.L., Yang X., Manickam S., Han Y. (2021). Comparison between airborne ultrasound and contact ultrasound to intensify air drying of blackberry: Heat and mass transfer simulation, energy consumption and quality evaluation. Ultrasonics Sonochemistry.

[bb0200] Tunc Y. (2025). Biochemical and antioxidant activities, organic acid contents, fatty acid compositions, mineral element contents, and carbohydrate contents of sumac (Rhus coriaria L.) accessions found in the eastern Mediterranean region of Türkiye. Journal of Food Composition and Analysis.

[bb0205] Usama M., Ali Z., Ndukwu M.C., Sathyamurthy R. (2023). The energy, emissions, and drying kinetics of three-stage solar, microwave and desiccant absorption drying of potato slices. Renewable Energy.

[bb0210] Wang D., Chen L., Ma Y., Zhang M., Zhao Y., Zhao X. (2019). Effect of UV-C treatment on the quality of fresh-cut lotus (Nelumbo nucifera Gaertn) root. Food Chemistry.

[bb0215] Wang H., Wang J., Mujumdar A.S., Jin X., Liu Z., Zhang Y., Xiao H. (2021). Effects of postharvest ripening on physicochemical properties, microstructure, cell wall polysaccharides contents (pectin, hemicellulose, cellulose) and nanostructure of kiwifruit (Actinidia deliciosa). Food Hydrocolloids.

[bb0220] Wang J., Chen Y.X., Wang H., Wang S.Y., Lin Z.N., Zhao L.L., Xu H.D. (2022). Ethanol and blanching pretreatments change the moisture transfer and physicochemical properties of apple slices via microstructure and cell-wall polysaccharides nanostructure modification. Food Chemistry.

[bb0225] Wang K.H., He P.Y., Wang Q.H., Yang Z.Q., Xing Y., Ren W.X., Xu H.D. (2024). Ultrasound pretreatment enhances moisture migration and drying quality of mulberry via microstructure and cell-wall polysaccharides nanostructure modification. Food Research International.

[bb0230] Wu X.F., Zhang M., Ye Y., Yu D. (2020). Influence of ultrasonic pretreatments on drying kinetics and quality attributes of sweet potato slices in infrared freeze drying (IRFD). LWT.

[bb0235] Xiao Y., Yang Y., Xu Y., Feng L., Nie M., Niu L., Liu C., Liu C., Li D., Yu Z. (2025). Ultrasonic pretreatment and drying temperature-induced modifications of three pectin fractions affect the microstructure and textural properties of dried grapes. Food Chemistry: X.

[bb0240] Xing Y., Wang K., Zhang M., Law C., Lei H., Wang J., Xu H. (2023). Pectin-interactions and the digestive stability of anthocyanins in thermal and non-thermal processed strawberry pulp. Food Chemistry.

[bb0245] Xu Y., Fan M., Ran J., Zhang T., Zheng H. (2015). Variation in phenolic compounds and antioxidant activity in apple seeds of seven cultivars. Saudi Journal of Biological Sciences.

[bb0250] Yang R.L., Li Q., Hu Q.P. (2020). Physicochemical properties, microstructures, nutritional components, and free amino acids of Pleurotus eryngii as affected by different drying methods. Scientific Reports.

[bb0255] Yeoh W.K., Ali A. (2017). Ultrasound treatment on phenolic metabolism and antioxidant capacity of fresh-cut pineapple during cold storage. Food Chemistry.

[bb0260] Zambrano-Zaragoza M.D.L.L., Mercado-Silva E., Gutierrez-Cortez E., Cornejo-Villegas M.A., Quintanar-Guerrero D. (2014). The effect of nano-coatings with α-tocopherol and xanthan gum on shelf-life and browning index of fresh-cut “red delicious” apples. Innovative Food Science & Emerging Technologies.

[bb0265] Zang Z., Huang X., Ma G., Wan F., Xu Y., Zhao Q., Liu Z. (2025). Novel edible coatings pretreatment for enhancing drying performance and physicochemical properties of cherry fruits during multi-frequency ultrasonic vacuum far infrared radiation-radio frequency vacuum segmented combination drying. Ultrasonics Sonochemistry.

[bb0270] Zhang J., Ding D., Lu J., Zhu J., Bai W., Guan P., Song Z., Chen H. (2024). Effect of electrohydrodynamic (EHD) drying on active ingredients, textural properties and moisture distribution of yam (Dioscorea opposita). Food Chemistry: X.

[bb0275] Zhang L., Liao L., Qiao Y., Wang C., Shi D., An K., Hu J. (2020). Effects of ultrahigh pressure and ultrasound pretreatments on properties of strawberry chips prepared by vacuum-freeze drying. Food Chemistry.

[bb0280] Zhang Q., Wan F., Zang Z., Jiang C., Xu Y., Huang X. (2022). Effect of ultrasonic far-infrared synergistic drying on the characteristics and qualities of wolfberry (*Lycium barbarum* L.). Ultrasonics Sonochemistry.

[bb0285] Zhang X., Wang Y., Nian R., Li Q., Zhu D., Cao X. (2024). Effects of ultrasonic pretreatment on drying characteristics and water migration characteristics of freeze-dried strawberry. Food Chemistry.

[bb0290] Zhang Y., Zhou L., Li Y., Meng C., Lei Y., Zhao H., Wang Y. (2015). Effect of radio frequency heating and blanching on physicochemical properties and microstructure of apple slices. Journal of Agricultural Science and Technology.

[bb0295] Zhao Y., Zhu H., Xu J., Zhuang W., Zheng B., Martin Lo Y., Huang Z., Tian Y. (2021). Microwave vacuum drying of lotus (Nelumbo nucifera Gaertn.) seeds: Effects of ultrasonic pretreatment on color, antioxidant activity, and rehydration capacity. LWT.

[bb0300] Zhu R., Jiang S., Li D., Law C.L., Han Y., Tao Y., Liu D. (2022). Dehydration of apple slices by sequential drying pretreatments and airborne ultrasound-assisted air drying: Study on mass transfer, profiles of phenolics and organic acids and PPO activity. Innovative Food Science and Emerging Technologies.

