# Supplementary material for: Integrated multi-dimensional micro-structure reveals the changes in moisture migration and quality attributes during drying of apple slices with ultrasonic pretreatment
Source: Food Chem X. 2025 Oct 21;31:103168. doi: 10.1016/j.fochx.2025.103168 (PMC12590438; doi:10.1016/j.fochx.2025.103168)

**Supplementary material**

**Table S1** Texture profiles of fresh apple tissues with ultrasonic treatment. US: ultrasonic

**Table S2** Equation of liner regression of 10 phenolic profiles and 4 organic acids.

**Table S3** Effects of different ultrasonic pretreatment and drying conditions on the color of apple slices.

Note: Different superscript letters in the same column reveal that there are significant differences under the same treatment (*P* < 0.05).

Figure S1. Original map of organic acids detected by HPLC in apples treated with different treatments. Note that the image only shows a set of parallel trial maps.

**Table S1**

| Parameters |  | Treatment |  |  |
| --- | --- | --- | --- | --- |
|  | US0 | US10 | US20 | US30 |
| Hardness (g) | 1713.83 ± 39.24^a^ | 1285.83 ± 54.33^b^ | 1136.36 ± 49.54^c^ | 1062.61 ± 41.69^c^ |
| Fracturability (g) | 1430.92 ± 43.80^a^ | 1290.87 ± 50.23^b^ | 1108.18 ± 39.70^c^ | 996.42 ± 59.80^d^ |
| Springiness (mm) | 0.71 ± 0.05^a^ | 0.61 ± 0.03^b^ | 0.59 ± 0.06^bc^ | 0.56 ± 0.04^c^ |
| Cohesiveness (g) | 0.20 ± 0.02^b^ | 0.20 ± 0.01^b^ | 0.22 ± 0.03^b^ | 0.26 ± 0.02^a^ |
| Gumminess (g) | 270.32 ± 34.01^ab^ | 231.87 ± 23.88^c^ | 240.65 ± 22.12^bc^ | 302.29 ± 24.15^a^ |
| Chewiness (mJ) | 272.61 ± 20.04^a^ | 158.41 ± 18.17^b^ | 139.12 ± 12.93^b^ | 137.52 ± 13.69^b^ |

Note: Different superscript letters in the same row reveal that there are significant differences under the same treatment (*P* < 0.05).

**Table S2**

| GroUS | Standard curve | Coefficient of determination (*R^2^*) |
| --- | --- | --- |
| Gallic acid | *y* = 3.0965 × 10^7^ *x* – 22398 | 0.9977 |
| Protocatechuic acid | *y* = 2.56277 × 10^7^ *x* – 4901 | 0.9955 |
| Chlorogenic acid | *y* = 2.07728 × 10^4^ *x* – 36504 | 0.9967 |
| Caffeic acid | *y* = 2.5014 × 10^7^ *x* + 54349 | 0.9827 |
| Epicatechin | *y* = 8.387364 × 10^6^ *x* + 15106 | 0.9989 |
| *p*-coumaric acid | *y* = 5.61335 × 10^7^ *x* + 21766 | 0.9998 |
| Ferulic acid | *y* = 7.623026 × 10^6^ *x* – 6480 | 0.9991 |
| Ellagic acid | *y* = 3.28859 × 10^7^ *x* – 38988 | 0.9990 |
| Phloridin | *y* = 2.1019 × 107 *x* – 45971 | 0.9995 |
| Quercetin | *y* = 2.00686 × 10^7^ *x* – 136758 | 0.9912 |
| Oxalic acid | *y* = 1.86291 × 10^7^ *x* + 95064 | 0.9993 |
| Citric acid | *y* = 1.507291 × 10^6^ *x* + 12399 | 0.9988 |
| Malic acid | *y* = 2.271842 × 10^6^ *x* + 3295 | 0.9982 |
| Fumaric acid | *y* = 1.92237 × 10^8^ *x –*360798 | 0.9996 |

**Table S3**

| Treatment | Color parameter | | | |
| --- | --- | --- | --- | --- |
|  | *L*^*^ | *a*^*^ | *b*^*^ | *ΔE* |
| US0+Raw | 87.86±0.22^a^ | 1.87±0.09^d^ | 21.56±0.16^d^ | / |
| US10+Raw | 86.93±0.02^b^ | 4.09±0.01^c^ | 22.43±0.31^c^ | 2.58±0.08^c^ |
| US20+Raw | 84.50±0.02^c^ | 4.56±0.11^b^ | 22.82±0.09^b^ | 4.49±0.07^b^ |
| US30+Raw | 81.28±0.07^d^ | 5.82±0.02^a^ | 23.91±0.02^a^ | 8.03±0.07^a^ |
| US0+HD50 | 84.17±0.01^c^ | 3.45±0.01^k^ | 24.43±0.03^e^ | 4.94 ±0.02^i^ |
| US10+HD50 | 74.95±0.12^i^ | 8.86±0.06^g^ | 26.57±0.09^c^ | 15.52±0.16^d^ |
| US20+HD50 | 73.51±0.04^k^ | 8.82±0.01^g^ | 27.45±0.03^a^ | 17.00±0.03^c^ |
| US30+HD50 | 70.92±0.01^l^ | 11.01±0.02^c^ | 26.87±0.02^b^ | 19.97±0.02^a^ |
| US0+HD60 | 85.52±0.02^b^ | 5.26±0.01^i^ | 20.93±0.01^i^ | 4.17 ±0.01^j^ |
| US10+HD60 | 78.00±0.03^e^ | 9.04±0.01^f^ | 22.92±0.02^f^ | 12.27±0.02^g^ |
| US20+HD60 | 79.61±0.01^d^ | 8.05±0.01^h^ | 22.87±0.01^f^ | 10.40±0.00^h^ |
| US30+HD60 | 75.08±0.01^h^ | 10.18±0.01^e^ | 25.06±0.02^d^ | 15.65±0.01^d^ |
| US0+HD70 | 86.16±0.01^a^ | 4.30±0.01^j^ | 22.52±0.02^g^ | 3.13 ±0.00^k^ |
| US10+HD70 | 77.67±0.03^f^ | 10.48±0.01^d^ | 19.87±0.15^a^ | 13.45±0.02^f^ |
| US20+HD70 | 74.77±0.03^j^ | 12.88±0.02^a^ | 18.87±0.03^k^ | 17.31±0.03^b^ |
| US30+HD70 | 76.80±0.01^g^ | 11.32±0.01^b^ | 21.27±0.01^h^ | 14.55±0.01^e^ |
| US0+IR50 | 87.68±0.02^b^ | 2.14±0.01^k^ | 27.39±0.02^c^ | 5.85 ±0.02^j^ |
| US10+IR50 | 80.14±0.03^f^ | 6.95±0.02^h^ | 25.83±0.03^d^ | 10.18±0.01^g^ |
| US20+IR50 | 79.02±0.02^g^ | 7.21±0.01^g^ | 29.98±0.03^b^ | 13.33±0.03^d^ |
| US30+IR50 | 80.32±0.02^f^ | 8.91±0.01^c^ | 20.83±0.01^g^ | 10.34±0.02^f^ |
| US0+IR60 | 87.19±0.02^c^ | 3.35±0.01^i^ | 23.06±0.02^f^ | 2.22 ±0.01^k^ |
| US10+IR60 | 81.66±0.01^e^ | 8.43±0.02^e^ | 22.98±0.03^f^ | 9.14 ±0.02^h^ |
| US20+IR60 | 77.93±0.02^h^ | 8.85±0.02^c^ | 31.03±0.01^a^ | 15.40±0.03^c^ |
| US30+IR60 | 79.02±0.01^b^ | 8.77±0.01^d^ | 23.75±0.02^e^ | 11.43±0.00^e^ |
| US0+IR70 | 89.26±0.01^a^ | 2.29±0.01^j^ | 20.58±0.01^h^ | 1.76 ±0.01^l^ |
| US10+IR70 | 82.33±0.02^d^ | 8.04±0.01^f^ | 20.27±0.02^i^ | 8.39 ±0.02^i^ |
| US20+IR70 | 77.47±0.02^i^ | 11.93±0.02^b^ | 14.90±0.02^k^ | 15.92±0.02^b^ |
| US30+IR70 | 74.75±0.26^j^ | 14.22±0.09^a^ | 18.15±0.14^j^ | 18.34±0.16^a^ |

Note: Different superscript letters in the same column reveal that there are significant differences under the same treatment (*P* < 0.05).

Figure S1.


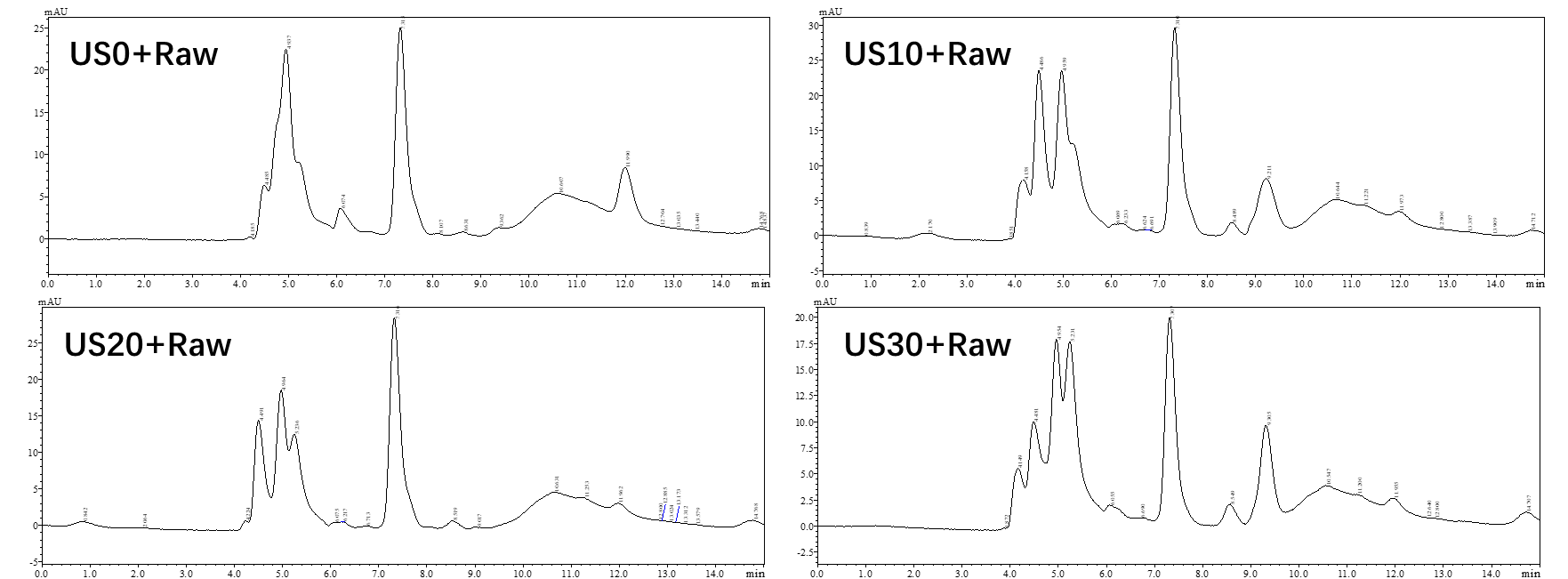


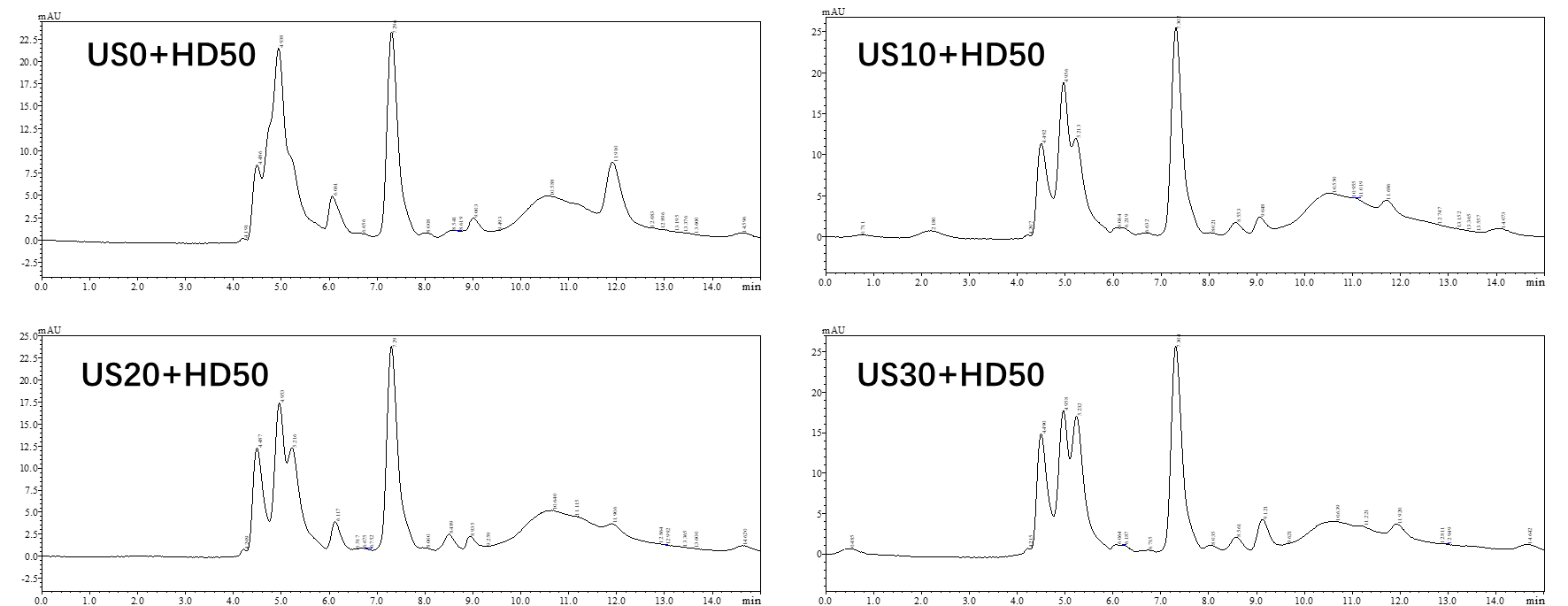


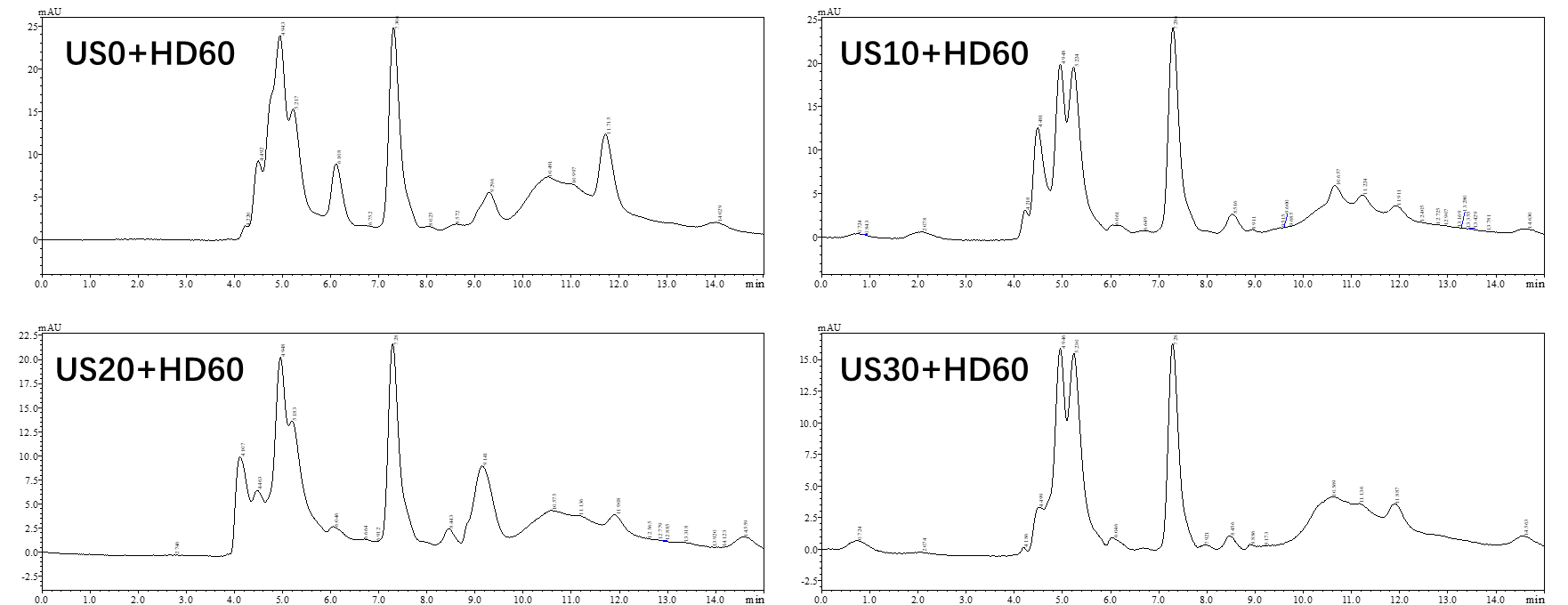


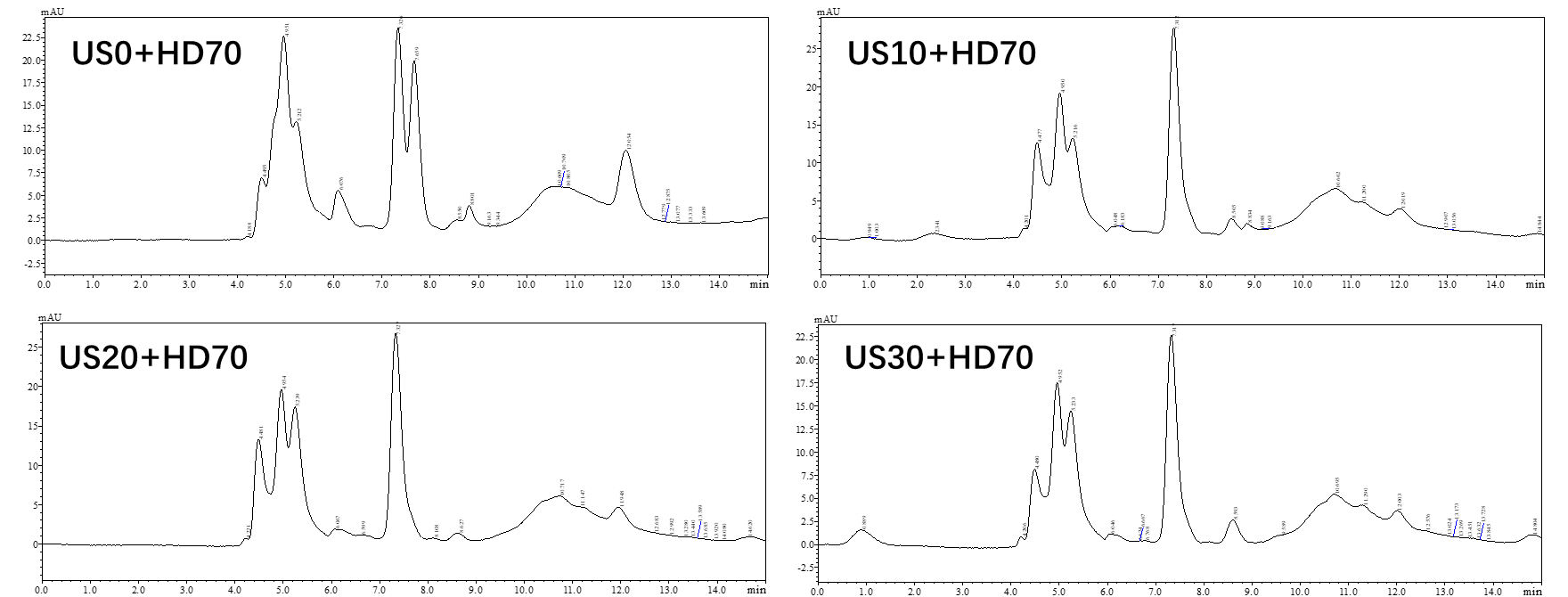


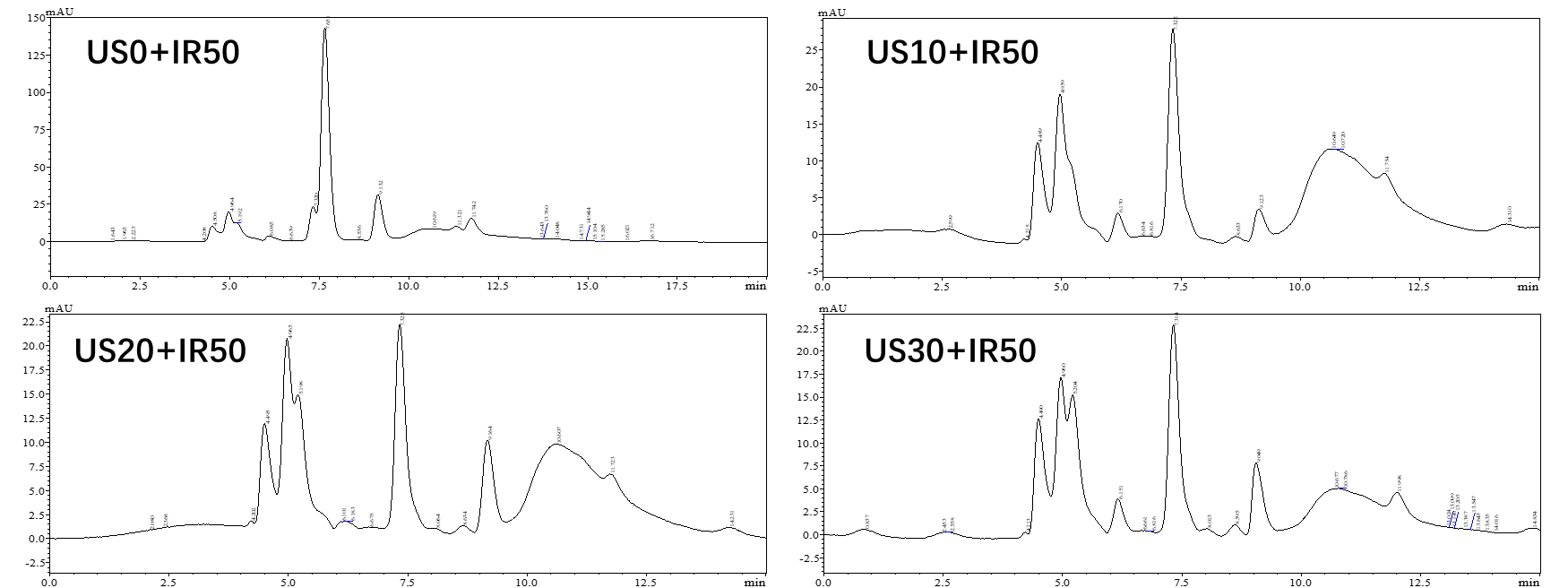


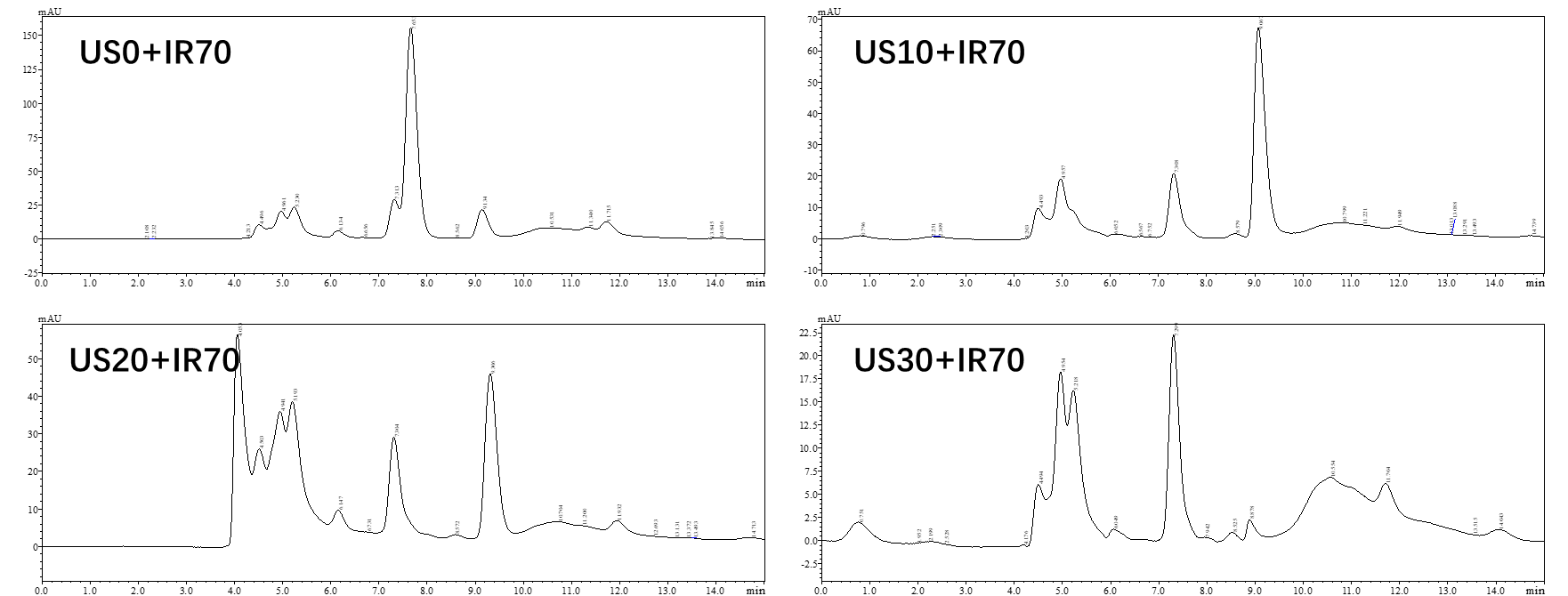

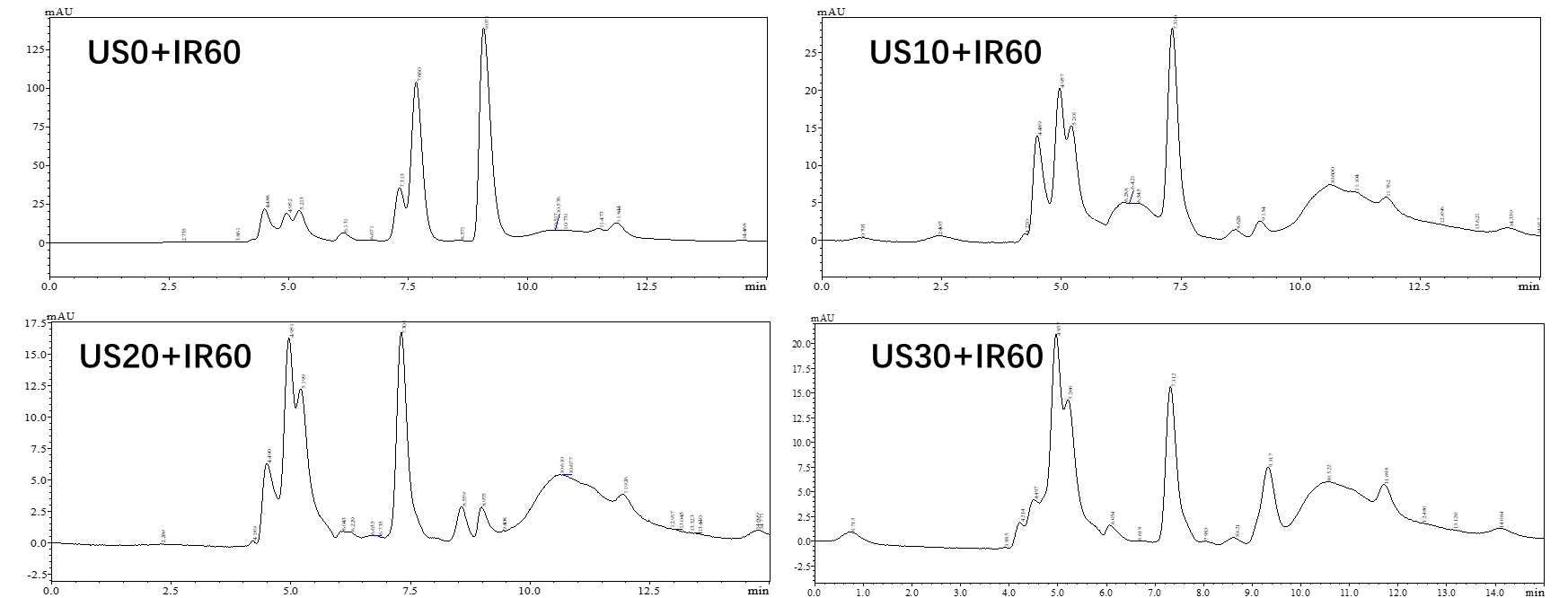

Supplement: Supplementary file 1 — Supplementary material [file mmc1.docx]
